# Cardiovascular effects of intravenous colforsin in normal and acute respiratory acidosis canine models: A dose-response study

**DOI:** 10.1371/journal.pone.0213414

**Published:** 2019-07-10

**Authors:** Takaharu Itami, Kiwamu Hanazono, Norihiko Oyama, Tadashi Sano, Kohei Makita, Kazuto Yamashita

**Affiliations:** 1 Department of Veterinary Medicine, Rakuno Gakuen University, Ebetsu, Hokkaido, Japan; 2 Department of Veterinary Science, Rakuno Gakuen University, Ebetsu, Hokkaido, Japan; University of PECS Medical School, HUNGARY

## Abstract

In acidosis, catecholamines are attenuated, and higher doses are often required to improve cardiovascular function. Colforsin activates adenylate cyclase in cardiomyocytes without beta-adrenoceptor. Here, six beagles were administered colforsin or dobutamine four times during eucapnia (partial pressure of arterial carbon dioxide 35–40 mm Hg; normal) and hypercapnia (ibid 90–110 mm Hg; acidosis) conditions. The latter was induced by CO_2_ inhalation. Anesthesia was induced with propofol and maintained with isoflurane. Cardiovascular function was measured by thermodilution and a Swan-Ganz catheter at baseline and 60 min after 0.3 μg/kg/min (low), 0.6 μg/kg/min (middle), and 1.2 μg/kg/min (high) colforsin administration. The median pH was 7.38 [range 7.33–7.42] and 7.01 [range 6.96–7.08] at baseline in the Normal and Acidosis conditions, respectively. Endogenous adrenaline and noradrenaline levels at baseline were significantly (*P* < 0.05) higher in the Acidosis than in the Normal condition. Colforsin induced cardiovascular effects similar to those caused by dobutamine. Colforsin increased cardiac output in the Normal condition (baseline: 3.9 ± 0.2 L/kg/m^2^ [mean ± standard error], low: 5.2 ± 0.4 L/kg/min^2^, middle: 7.0 ± 0.4 L/kg/m^2^, high: 9.4 ± 0.2 L/kg/m^2^; *P* < 0.001) and Acidosis condition (baseline: 6.1 ± 0.3 L/kg/m^2^, low: 6.2 ± 0.2 L/kg/m^2^, middle: 7.2 ± 0.2 L/kg/m^2^, high: 8.3 ± 0.2 L/kg/m^2^; *P* < 0.001). Colforsin significantly increased heart rate and decreased systemic vascular resistance compared to values at baseline. Both drugs increased pulmonary artery pressure, but colforsin (high: 13.3 ± 0.6 mmHg in Normal and 20.1 ± 0.2 mmHg in Acidosis) may have lower clinical impact on the pulmonary artery than dobutamine (high: 19.7 ± 0.6 in Normal and 26.7 ± 0.5 in Acidosis). Interaction between both drugs and experimental conditions was observed in terms of cardiovascular function, which were similarly attenuated with colforsin and dobutamine under acute respiratory acidosis.

## Introduction

Colforsin daropate is a forskolin derivative that directly activates adenylate cyclase in cardiomyocytes and vascular smooth muscle without mediating the catecholamine beta-adrenoceptor. As with dobutamine, a catecholamine beta agonist, colforsin increases cardiac contractility and reduces peripheral vascular resistance [1,2]. When forskolin was first discovered, it was poorly soluble in water, and its clinical application as an injection was limited. Colforsin was prepared as a water-soluble forskolin derivative and became available in 1999 [3].

Colforsin has been tested on human patients with congestive heart failure, and it improved their hemodynamics [3]. Severe heart failure with pulmonary edema often complicates respiratory acidosis. In acidosis, myocardial beta-1 adrenoceptor is downregulated. Therefore, catecholamine responses to decreases in cAMP decline. For this reason, cardiac contractility is suppressed in sepsis and acidosis [4]. Unlike adrenaline, colforsin improved cardiac function in rat cardiac resection specimens even under acidosis [5]. However, the efficacy of colforsin was never compared with that of catecholamines, and no investigation has been conducted on the effects of colforsin in living organisms under acidosis. We hypothesized that colforsin maintains cardiac function in acidotic dogs.

The aims of this study were to examine the cardiovascular effects of colforsin in an acute respiratory acidosis model induced by carbon dioxide inhalation.

## Materials and methods

### Experimental animals

Six beagles (3 females, 3 males) aged 1–2 y and weighing 9.5–12.5 kg were used in this study. The dogs were judged to be in good health based on the results of physical examinations, complete blood cell counts, and serum biochemical analyses. The dogs were owned by the university and maintained according to the principles of the “Guide for the Care and Use of Laboratory Animals” prepared by Hokkaido University and approved by the Association for Assessment and Accreditation of Laboratory Animal Care International (AAALAC). The dogs were raised in a cage without a ceiling of at least 0.74 m^2^, and exercised daily. Each dog was fed with an appropriate amount of food twice a day, and their health status was monitored daily by a dedicated veterinarian. The Animal Care and Use Committee of Hokkaido University approved the study (No. 14–0156). Food (but not water) was withheld from the dogs for 12 h before the experiment day. Dogs in normal and acidotic condition were administered colforsin or dobutamine. Each dog was anesthetized 4× at 2-week intervals. This study was performed using a random number table under a randomized crossover design.

### Experimental preparations

All dogs were fitted with 22-gauge catheters (Surflow; Terumo Co. Ltd., Tokyo, Japan) in both cephalic veins and administered 6 mg/kg propofol (Propoflo 28; Zoetis Co. Ltd., Tokyo, Japan) intravenously through a catheter placed in the right cephalic vein. They were orotracheally intubated and connected to a standard circle anesthesia system (FO-20A; ACOMA Medical Industry Co. Ltd., Tokyo Japan) and a ventilator (Spiritus; ACOMA Medical Industry Co. Ltd., Tokyo Japan). All dogs received Ringer’s solution (Fuso Pharmaceutical Industries Ltd., Osaka, Japan) at 5 mL/kg/h and vecuronium (Fuji Pharma Co. Ltd., Tokyo, Japan) by injection at 0.1 mg/kg. They were then intravenously infused with 0.1 mg/kg/h vecuronium through a catheter in the right cephalic vein to prevent reflex respiratory muscle movement. The dogs were mechanically ventilated with oxygen and received 1.3–1.5% end-tidal isoflurane anesthesia. The oxygen flow rate was 2 L/min. They were placed in left lateral recumbency and mechanically ventilated at a respiratory rate of 12 breaths/min and a 1:2 inspiratory-expiratory ratio with volume control ventilation (tidal volume = 9–13 mL/kg) without using positive end-expiratory pressure and end-expiratory hold.

A 22-gauge catheter was inserted percutaneously into a left dorsal pedal artery. Three pressure transducers (DT-NN; Merit Medical Co. Ltd., Tokyo, Japan) were prepared and calibrated against a mercury manometer at 200 mm Hg, 50 mm Hg, and 20 mm Hg for the mean arterial, pulmonary arterial, and right atrial pressures, respectively. The right neck region was shaved and aseptically prepared. Approximately 0.5 mL of 2% lidocaine (Xylocaine; Astra-Zeneca, Osaka, Japan) was injected subcutaneously. A 5-Fr, 75-cm Swan-Ganz catheter (132F5; Edwards Lifesciences Co. Ltd., Tokyo, Japan) was inserted into a jugular vein using a 6-Fr introducer (Medikit Catheter Introducer; Medikit Co. Ltd., Tokyo, Japan). The distal port of the Swan-Ganz catheter was connected to a pressure transducer and advanced into the pulmonary artery using the characteristic pressure changes associated with the right ventricle and pulmonary artery. A transducer was attached to the arterial catheter to measure mean arterial pressures (MAP; mm Hg). Transducers were connected to the distal and proximal ports of the Swan-Ganz catheter to measure mean pulmonary arterial pressure (mPAP; mm Hg) at the distal port, pulmonary arterial occlusion pressure (PAOP; mm Hg) at the distal port, and mean right atrial pressure (RAP; mm Hg) at the proximal port. All pressure transducers were zeroed at the mid-sternum level. The PAOP was measured after distal balloon inflation on the Swan-Ganz catheter at the end of expiration.

Cardiac output (CO; L/min) was determined by thermodilution. Five milliliters of normal saline (1–4 °C) was rapidly injected manually into the proximal port of the Swan-Ganz catheter at the end of expiration. Temperature fluctuations were measured with a thermosensor placed at the tip of the Swan-Ganz catheter. At each time interval, three consecutive measurements within 10% of each other were recorded, and the average was recorded as the CO. The thermistor on the Swan-Ganz catheter measured the core body temperature, which was maintained between 37.0–37.5 °C by a forced-air patient-warming machine (Bair Hugger; 3M Japan Co. Ltd., Tokyo, Japan).

After the dogs were instrumented, the normal and acidotic conditions were adjusted according to the arterial blood gas data. Arterial- and mixed venous blood gases were measured by collecting 1.0 mL blood from the dorsal pedal- and pulmonary arteries catheterized to a heparinized syringe. Blood gas measurements (GEM-Premier 3000; IL Japan Co. Ltd., Tokyo, Japan) were corrected to body temperature. When the cardiovascular parameters were being measured in the normal condition (Normal), the arterial partial pressure of carbon dioxide (PaCO_2_) was maintained at ~35–40 mm Hg. When the cardiovascular parameters were being measured in the acidotic condition (Acidosis), the PaCO_2_ was maintained at ~90–110 mm Hg, and the pH was ~7.0. Exogenous hypercapnia was induced by adding dry gaseous carbon dioxide (CO_2_) to the inspiratory corrugated tube of the anesthesia circuit.

### Evaluation of cardiovascular parameters

All dogs were stabilized for 30 min after preparation. Then, baseline cardiovascular parameters and arterial- and mixed venous blood gases were measured and recorded as follows: heart rate (HR; beats/min) by electrocardiogram with a lead II, and MAP, mPAP, RAP, and PAOP by a multi-parameter anesthetic monitoring system (RMC-4000 Cardio Master; Nihon Kohden Corporation, Tokyo, Japan). Cardiac index (CI; mL/min/m^2^), stroke volume (SVI; mL/beat/m^2^), systemic vascular resistance (SVRI; dynes·sec·cm^-5^/ m^2^), and pulmonary vascular resistance (PVRI; dynes·sec·cm^-5^/ m^2^) were calculated by inserting values into previously published formulae [6].

**Fig 1:**
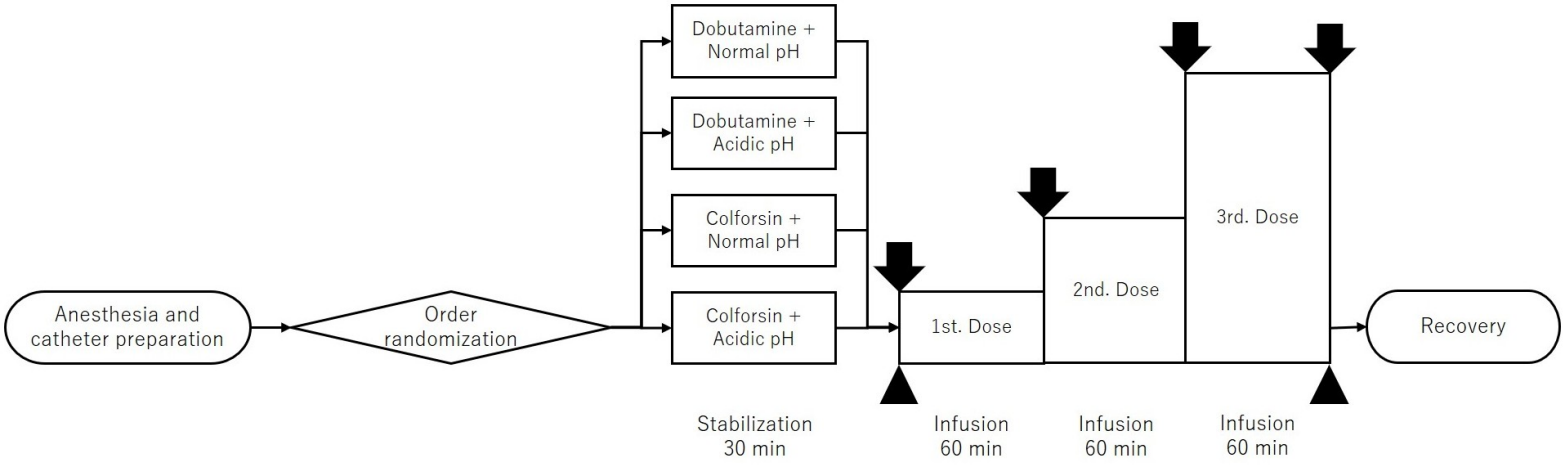
Time course of study regimen. After anesthesia and catheter preparation, four experimental sequences were randomized using a random number table, and then the dogs were stabilized for 30 min in each condition. Colforsin (1st. Dose: 0.3 μg/kg/min, 2nd. Dose: 0.6 μg/kg/min, 3rd. Dose: 1.2 μg/kg/min) and dobutamine (1st. Dose: 5 μg/kg/min, 2nd. Dose: 10 μg/kg/min, 3rd. Dose: 20 μg/kg/min) were infused 60 min. Cardiovascular parameters were measured at baseline and the end of each increment dose. The arrow indicates the measurement of cardiovascular parameters. The arrow-head indicates the measurement of blood gas and biochemical test.

After the baseline measurements, the dogs were intravenously infused with colforsin (Adehl; Nihonkayaku Co. Ltd., Tokyo, Japan) or dobutamine (Dobutrex; Shionogi & Co. Ltd., Osaka, Japan) through a 22-gauge catheter inserted into the left cephalic vein. Colforsin administration was gradually increased to 1 mL/h (0.3 μg/kg/min), 2 mL/h (0.6 μg/kg/min), and 4 mL/h (1.2 μg/kg/min) every 60 min. Similarly, dobutamine administration was gradually increased to 1 mL/h (5 μg/kg/min), 2 mL/h (10 μg/kg/min), and 4 mL/h (20 μg/kg/min) every 60 min. Colforsin and dobutamine were diluted with sterile saline (normal saline; Otsuka Pharmaceutical Factory Inc., Tokyo, Japan) and administered by an infusion pump (TOP-5500; TOP Co. Ltd., Tokyo, Japan). All cardiopulmonary measurements were repeated every 60 min after each dose was administered. When the cardiovascular parameters were determined after the final dose, the arterial- and mixed venous blood gases were measured as described above (Fig 1).

After the experiment, all dogs received 0.2 mg/kg subcutaneous meloxicam (Metacam; Boehringer Ingelheim Co. Ltd., Tokyo, Japan) and 0.01 mg/kg intramuscular buprenorphine (Lepetan injection; Otsuka Pharmaceutical Factory Inc., Tokyo, Japan) for analgesia and 25 mg/kg intravenous cefazolin (cefazolin sodium; Nichi-Iko Co. Ltd., Toyama, Japan) to prevent infection. For the Acidosis condition, carbon dioxide inhalation was terminated, and dog PaCO_2_ was maintained at 35–40 mm Hg. They were administered 0.5 g/kg intravenous mannitol (*D*-mannitol injection; Terumo Co. Ltd., Tokyo, Japan) for 30 min to lower intracranial pressure. Colforsin and dobutamine were washed out for 1 h and the dogs recovered from the anesthesia.

### Biochemical examination

Two milliliters of blood was drawn from the arterial catheter to measure baseline catecholamine (adrenaline, noradrenaline, and dopamine) concentrations. The obtained blood samples were collected into an ethylenediaminetetraacetic acid (EDTA) tube and immediately centrifuged (1,000 × g for 10 min at 4 °C) to separate the plasma, which was then stored at -80 °C until analysis. The frozen plasma was sent to an external commercial laboratory (BML Inc., Tokyo, Japan) to determine catecholamine levels. In addition, 2 mL blood was drawn from the arterial catheter and collected into a lithium heparinized tube at baseline and at the end of the experiment, the plasma was then isolated as described above, and the samples were immediately biochemically analyzed (DRI-CHEM 7000V; Fujifilm Co. Ltd., Tokyo, Japan) without freezing in the laboratory at our facility. This analyzer was inspected every day before operation and used to measure blood glucose, blood urea nitrogen, creatinine, and electrolyte (S1 File).

### Statistical analysis

The data were processed using statistical software (BellCurve for Excel; Social Survey Research Information Co. Ltd., Tokyo, Japan) and online in R v. 3.5.3. (2019-03-11). A Wilcoxson signed-rank test was used to compare biochemical and blood gas measurements between baseline and the end of the experiment. Cardiovascular variables were confirmed for the Shapiro-Wilk normality test, and their baseline values at normal and acidotic condition were compared using a linear mixed-effects model fit by restricted maximum likelihood (R with package nlme). The same package was also used to compare cardiovascular variables at each colforsin and dobutamine dose between the Normal and Acidosis conditions. Differences were considered significant when two-sided *P* < 0.05.

## Results

### Blood gases and biochemical analyses

The blood gas and biochemical test results at baseline are shown in Table 1. The median pH at baseline was 7.38 (range 7.33–7.42) for the Normal condition and 7.01 (6.96–7.08) for the Acidosis condition. The PaCO_2_ at baseline was 39 mm Hg (range 34–42 mm Hg) for the Normal condition and 101 mm Hg (range 92–114 mm Hg) for the Acidosis condition. PaCO_2_ slightly increased during the experiment, and the pH slightly decreased by the end of the experiment. Blood adrenaline and noradrenaline levels were significantly higher in the Acidosis than in the Normal condition at baseline. Plasma glucose level was also significantly higher in the Acidosis condition than the Normal condition. Plasma potassium was significantly higher at the end of the experiment than it was at baseline. No clinically significant changes were observed in the other variables in this study (S1 Table).

**Table 1:** The blood gas examination and blood biochemical tests in six anesthetized dogs in eucapnia (Normal) and acute respiratory acidosis (Acidosis) at baseline.

Variable (Unit)	Normal	Acidosis	Reference
Adrenaline (ng/mL	0.01 [0.01–0.13]	0.15 [0.05–2.08]*	<0.10
Noradrenaline (ng/mL)	0.04 [0.01–0.46]	0.33 [0.17–0.97]*	0.10~0.50
Dopamine (ng/mL)	0.01 [0.01–0.04]	0.02 [0.01–0.08]	<0.03
PCV (%)	28 [24–39]	39 [29–49]	37–55
pH	7.38 [7.33–7.42]	7.01 [6.96–7.08]*	7.35–7.45
PaCO_2_ (mm Hg)	39 [34–42]	101 [92–114]*	30.8–42.8
PaO_2_ (mm Hg)	547 [495–616]	522 [443–551]	80.9–103.3
HCO_3_^-^ (mEq/L)	22.8 [20.8–25.4]	26.5 [24.6–27.9]*	18.8–25.6
BEecf (mEq/L)	-2.0 [-4.5–1.0]	-5.0 [-7.0–-2.6]	-4–+4
Lactate (mmol/L)	1.5 [0.6–3.2]	0.5 [0.3–1.0]*	<2.0
Na (mEq/L)	144 [142–148]	146 [142–150]	135–147
K (mEq/L)	3.8 [3.2–4.3]	3.9 [3.2–4.3]	3.5–5.0
Cl (mEq/L)	115 [111–119]	115 [109–118]	95–125
Glucose (mg/dL)	103 [89–137]	140 [114–200]*	60–110
BUN (mg/dL)	13.7 [9.0–17.5]	15.5 [11.0–22.0]	10–20
Creatinine (mg/dL)	0.5 [0.3–0.9]	0.6 [0.4–0.7]	0.6–1.2

Data show median (range). PCV, packed cell volume; PaCO_2_, arterial partial pressure of carbon dioxide; PaO_2_, arterial partial pressure of oxygen; HCO_3_^-^, bicarbonate ion; BEecf, base excess in the extracellular fluid; Na, sodium ion; K, potassium ion, Cl, chloride ion; BUN, blood urea nitrogen. The reference values were shown from individual testing apparatus.

* shows significant difference (*P* < 0.05) from baseline in Normal condition by Wilcoxson signed-rank test.

### Cardiovascular effects of colforsin and dobutamine

**Fig 2:**
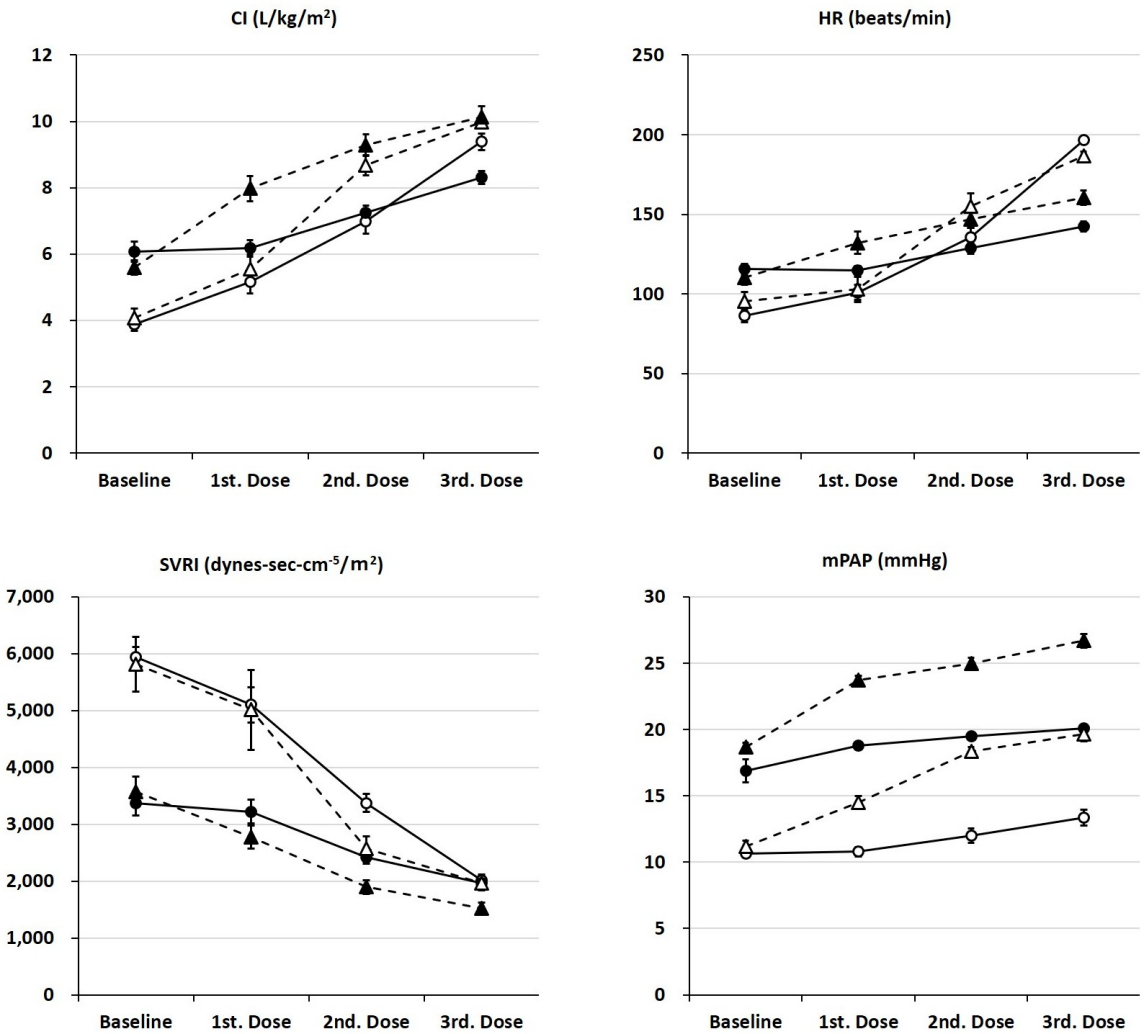
The effects of colforsin and dobutamine on cardiac output, heart rate, systemic vascular resistance, and mean pulmonary artery pressure in normal and acidotic condition. Upper left: cardiac index (CI), Upper right: heart rate (HR), Lower left: systemic vascular resistance index (SVRI), Lower right: mean pulmonary artery pressure (mPAP). In each graph, the horizontal axis shows the dosage of colforsin (Baseline, 1st. Dose: 0.3 μg/kg/min, 2nd. Dose: 0.6 μg/kg/min, 3rd. Dose: 1.2 μg/kg/min) and dobutamine (Baseline, 1st. Dose: 5 μg/kg/min, 2nd. Dose: 10 μg/kg/min, 3rd. Dose: 20 μg/kg/min). The white and black circle shows colforsin in normal and acidotic condition, respectively. The white and black triangle shows dobutamine in normal and acidotic condition, respectively. The plot and error bar shows mean value and standard error, respectively.

In the Normal condition, the CI, HR, and mPAP were significantly higher, and SVRI was lower at baseline as compared to the Acidosis condition (P < 0.001 in all). The effects of colforsin and dobutamine on CI, HR, SVRI, and mPAP in the Normal and Acidosis conditions are shown in Fig 2. Colforsin and dobutamine dose-dependently increased CI, HR, mPAP, and decreased SVRI in Normal and Acidosis condition (P < 0.001 in all). Interaction between both drugs and condition was observed in CI, HR, and SVRI, excluding mPAP (Table 2 and S2 File). In colforsin administration, relative to the baseline value, the rate of increase in CI in the Acidosis condition was attenuated as compared with the respective values in the Normal condition (Normal vs. Acidosis: 133% vs. 102% at 0.3 μg/kg/min; 180% vs. 119% at 0.6 μg/kg/min; 242% vs. 137% at 1.2 μg/kg/min). In case of dobutamine administration, relative to the baseline value, the rate of increase in CI in the Acidosis condition was attenuated as compared with the Normal condition (Normal vs. Acidosis: 136% vs. 142% at 5 μg/kg/min; 213% vs. 166% at 10 μg/kg/min; 245% vs. 181% at 20 μg/kg/min). The effects of colforsin and dobutamine on the other cardiovascular parameters in the Normal and Acidosis conditions are shown in Tables 3 and 4, and S2 Table. In colforsin administration, the numbers of dogs with mPAP > 20 mm Hg were one (17%) at baseline, zero (0%) at 0.3 μg/kg/min, one (17%) at 0.6 μg/kg/min, and one (17%) at 1.2 μg/kg/min in the Acidosis condition. In dobutamine administration, the numbers of dogs with mean PAP > 20 mm Hg were two (33%) at 10 μg/kg/min and three (50%) at 20 μg/kg/min in the Normal condition, zero (0%) at baseline, and six (100%) > 5 μg/kg/min in the Acidosis condition. Atrial stasis was observed by the end of the experiment in one acidotic dog receiving dobutamine. Its plasma potassium was 7.5 mmol/L. After the experiment, its cardiac rhythm reverted to a normal electrocardiogram waveform. No other arrhythmia was observed.

**Table 2:** Linear mixed-effects model results for the effects of colforsin/dobutamine and pH condition on the cardiac index.

Variables	Estimate	Standard error	*P*-value
Colforsin			
Intercept	3.98	0.49	<0.001
Fixed effects			
Dose of colforcin	4.63	0.18	<0.001
Acidosis (normal as reference)	1.98	0.18	<0.001
Dose: acidosis (normal as reference)	-2.63	0.25	<0.001
Random effect	Variance	Variance of residual	ICC%*
Dogs	1.38	0.56	71.3
Variables	Estimate	Standard error	*P*-value
Dobutamine			
Intercept	4.40	0.52	<0.001
Fixed effects			
Dose of dobutamine	0.30	0.02	<0.001
Acidosis (normal as reference)	1.95	0.26	<0.001
Dose: acidosis (normal as reference)	-0.08	0.02	<0.001
Random effect	Variance	Variance in residual	ICC%*
Dogs	1.44	1.03	58.3

*ICC: Intra class correlation percentage: variance of random effect / total variance

**Table 3:** Mean ± standard error values for cardiovascular variables at baseline, 0.3 μg/kg^/^min, 0.6 μg/kg^/^min, and 1.2 μg/kg^/^min dose of intravenous colforsin in six anesthetized dogs in eucapnia (Normal) and acute respiratory acidosis (Acidosis) conditions.

Variable (Unit)	Condition	Baseline	1st. Dose	2nd. Dose	3rd. Dose	*P*-value		
			0.3 μg/kg/min	0.6 μg/kg/min	1.2 μg/kg/min	Condition	Treatment	Interaction
CI (L/min/m^2^)	Normal	3.9 ± 0.2	5.2 ± 0.4	7.0 ± 0.4	9.4 ± 0.2	<0.001	<0.001	<0.001
	Acidosis	6.1 ± 0.3*	6.2 ± 0.2	7.2 ± 0.2	8.3 ± 0.2			
HR (beats/min)	Normal	86.0 ± 3.9	100.9 ± 4.6	135.4 ± 6.2	196.4 ± 1.5	<0.001	<0.001	<0.001
	Acidosis	115.5 ± 3.2*	114.6 ± 2.9	128.5 ± 3.3	142.2 ± 3.2			
SVI (mL/beat/m^2^)	Normal	43.5 ± 2.4	48.0 ± 1.6	49.4 ± 1.7	45.3 ± 1.2	<0.001	0.510	0.034
	Acidosis	52.2 ± 1.5*	53.7 ± 1.4	56.3 ± 0.8	58.6 ± 1.0			
SAP (mm Hg)	Normal	96.1 ± 3.5	100.1 ± 2.9	92.3 ± 2.1	79.9 ± 1.8	0.016	<0.001	0.764
	Acidosis	93.2 ± 1.3	90.4 ± 1.5	83.8 ± 3.3	76.4 ± 3.7			
MAP (mm Hg)	Normal	68.5 ± 2.6	72.4 ± 2.3	65.5 ± 1.6	55.5 ± 2.1	0.010	<0.001	0.216
	Acidosis	66.4 ± 1.9	66.5 ± 1.6	60.5 ± 2.6	56.7 ± 2.8			
DAP (mm Hg)	Normal	54.2 ± 2.0	58.4 ± 2.4	54.7 ± 1.2	50.2 ± 1.6	<0.001	0.006	0.172
	Acidosis	50.5 ± 1.6	47.0 ± 1.1	44.2 ± 1.8	41.0 ± 1.7			
RAP (mm Hg)	Normal	3.8 ± 0.3	3.3 ± 0.3	3.0 ± 0.2	2.4 ± 0.2	<0.001	<0.001	0.091
	Acidosis	3.9 ± 0.2	5.3 ± 0.1	4.1 ± 0.1	3.6 ± 0.2			
SVRI (dynes・sec・cm^-5^/m^2^)	Normal	5,946 ± 173	5,100 ± 309	3,376 ± 155	2,014 ± 98	<0.001	<0.001	<0.001
	Acidosis	3,367 ± 217*	3,221 ± 210	2,417 ± 111	1,965 ± 109			
mPAP (mm Hg)	Normal	10.7 ± 0.2	10.8 ± 0.4	12.0 ± 0.6	13.3 ± 0.6	<0.001	<0.001	0.9598
	Acidosis	16.9 ± 0.9*	18.8 ± 0.1	19.5 ± 0.2	20.1 ± 0.2			
PAOP (mm Hg)	Normal	4.5 ± 0.2	4.0 ± 0.1	3.4 ± 0.1	3.3 ± 0.3	<0.001	<0.01	0.018
	Acidosis	10.9 ± 0.2*	9.8 ± 0.5	8.7 ± 0.3	8.4 ± 0.2			
PVRI (dynes・sec・cm^-5^/m^2^)	Normal	607.2 ± 52.3	491.8 ± 32.3	454.7 ± 32.0	379.4 ± 28.6	<0.001	<0.001	<0.001
	Acidosis	395.0 ± 18.1*	467.6 ± 34.2	483.5 ± 28.6	442.4 ± 15.6			

CI, cardiac index; HR, heart rate; SVI, stroke volume index; SAP, systolic arterial pressure; MAP, mean arterial pressure, DAP, diastolic arterial pressure; RAP, right atrial pressure; SVRI, systemic vascular resistance index; mPAP, mean pulmonary arterial pressure; PAOP, pulmonary arterial occlusion pressure; PVRI, pulmonary vascular resistance index. Linear mixed-effects model applies on the condition and dobutamine treatment.

* shows a significant difference (*P* < 0.05) as compared with Normal condition at baseline. Body surface area (m^2^) = 10.1 × Body weight (kg)^0.67^ / 100

**Table 4:** Mean ± standard error values for cardiovascular variables at baseline, 5 μg/kg^/^min, 10 μg/kg^/^min, and 20 μg/kg^/^min dose of intravenous dobutamine in six anesthetized dogs in eucapnia (Normal) and acute respiratory acidosis (Acidosis) conditions.

Variable (Unit)	Condition	Baseline	1st. Dose	2nd. Dose	3rd. Dose	*P*-value		
			5 μg/kg/min	10 μg/kg/min	20 μg/kg/min	Condition	Treatment	Interaction
CI (L/min/m^2^)	Normal	4.1 ± 0.3	5.5 ± 0.4	8.7 ± 0.3	10.0 ± 0.2	<0.001	<0.001	<0.001
	Acidosis	5.6 ± 0.2*	8.0 ± 0.4	9.3 ± 0.3	10.1 ± 0.3			
HR (beats/min)	Normal	95.1 ± 6.0	102.9 ± 7.9	154.9 ± 8.2	186.6 ± 2.7	<0.001	<0.001	<0.001
	Acidosis	110.1 ± 4.3*	132.0 ± 7.0	146.9 ± 5.5	160.6 ± 4.5			
SVI (mL/beat/m^2^)	Normal	42.7 ± 1.2	53.8 ± 1.6	57.2 ± 1.6	53.5 ± 0.9	<0.001	<0.001	0.610
	Acidosis	51.2 ± 1.3*	61.3 ± 2.1	63.7 ± 1.6	63.3 ± 1.5			
SAP (mm Hg)	Normal	93.7 ± 2.1	97.7 ± 3.2	88.2 ± 3.5	79.7 ± 3.4	0.459	<0.001	0.081
	Acidosis	94.8 ± 2.3	100.6 ± 2.2	84.3 ± 3.5	73.1 ± 4.1			
MAP (mm Hg)	Normal	68.1 ± 2.2	69.4 ± 2.3	64.7 ± 2.3	58.8 ± 1.9	0.260	0.001	0.053
	Acidosis	69.8 ± 2.4	73.5 ± 2.2	61.7 ± 2.1	53.9 ± 2.2			
DAP (mm Hg)	Normal	54.7 ± 2.2	52.8 ± 1.9	48.0 ± 1.5	44.0 ± 1.1	0.974	<0.001	0.367
	Acidosis	51.7 ± 1.5	56.0 ± 1.9	46.7 ± 1.4	40.3 ± 1.4			
RAP (mm Hg)	Normal	3.0 ± 0.3	3.0 ± 0.3	2.8 ± 0.3	2.5 ± 0.2	<0.001	0.028	0.574
	Acidosis	5.0 ± 0.2*	4.6 ± 0.4	4.5 ± 0.4	4.6 ± 0.4			
SVRI (dynes・sec・cm^-5^/m^2^)	Normal	5,818.2 ± 480.2	5,013.0 ± 703.7	2,567.0 ± 218.5	1,959.1 ± 119.8	<0.001	<0.001	<0.001
	Acidosis	3,572.0 ± 266.1*	2,778.9 ± 202.7	1,896.7 ± 115.5	1,527.8 ± 94.8			
mPAP (mm Hg)	Normal	11.2 ± 0.5	14.5 ± 0.5	18.3 ± 0.4	19.7 ± 0.6	<0.001	<0.001	0.120
	Acidosis	18.7 ± 0.3*	23.7 ± 0.3	25.0 ± 0.4	26.7 ± 0.5			
PAOP (mm Hg)	Normal	4.7 ± 0.3	4.8 ± 0.4	4.7 ± 0.2	4.3 ± 0.3	<0.001	0.173	0.425
	Acidosis	9.8 ± 0.5*	10.5 ± 0.4	10.0 ± 0.4	9.9 ± 0.3			
PVRI (dynes・sec・cm^-5^/m^2^)	Normal	569.9 ± 32.2	656.6 ± 57.4	549.3 ± 31.6	530.2 ± 31.5	<0.001	0.009	0.063
	Acidosis	486.5 ± 30.1*	537.9 ± 39.8	505.6 ± 35.0	519.8 ± 30.5			

CI, cardiac index; HR, heart rate; SVI, stroke volume index; SAP, systolic arterial pressure; MAP, mean arterial pressure, DAP, diastolic arterial pressure; RAP, right atrial pressure; SVRI, systemic vascular resistance index; mPAP, mean pulmonary arterial pressure; PAOP, pulmonary arterial occlusion pressure; PVRI, pulmonary vascular resistance index. Linear mixed-effects model applies on the condition and dobutamine treatment.

* shows a significant difference (*P* < 0.05) as compared with Normal condition at baseline. Body surface area (m^2^) = 10.1 × Body weight (kg)^0.67^ / 100

Miosis was observed in three dogs (50%) receiving colforsin and four dogs (67%) receiving dobutamine in the Acidosis condition but it disappeared within 6 h after the end of colforsin infusion. In recovery period, nausea or vomiting was transiently observed in one dog (17%) in the Normal condition and two dogs (33%) in the Acidosis condition receiving colforsin infusion, and in three dogs (50%) in the Normal condition and two dogs (33%) in the Acidosis condition receiving dobutamine infusion. All dogs ate and drank within 3 h after the end of the experiment. No dog presented with complications as a result of the drug administration according to their blood chemistry and general physical examinations 2 weeks after the end of the experiment.

## Discussion

To the best of our knowledge, this study is the first to evaluate the dose-dependent cardiovascular function of colforsin in dogs. Colforsin had a cardiovascular action similar to that of dobutamine. It increased CI and HR and decreased SVRI in a dose-dependent manner. Both drugs also increased pulmonary artery pressure, but colforsin may have a lower clinical impact on pulmonary artery than dobutamine. However, under acute respiratory acidosis, CI, HR, and SVRI were attenuated with both colforsin and dobutamine. Interaction between both drugs and experimental conditions was observed in terms of cardiovascular function, which were similarly attenuated with colforsin and dobutamine under acute respiratory acidosis.

Dobutamine is a synthetic dopamine analog which stimulates beta-1, beta-2, and alpha-1 adrenoceptors in the cardiovascular system at doses approximating those used clinically (1–20 μg/kg/min) [7,8]. The inotropic activity of dobutamine is the result of stimulating both beta-1 and alpha-1 adrenoceptors in the myocardium. Furthermore, the beta-2 adrenoceptor-mediated vasodilatory effect of dobutamine is offset by alpha-1 adrenoceptor-mediated vasoconstrictor activity. Therefore, dobutamine increases CI and HR and decreases SVRI (inodilation) in a dose-dependent manner [9,10]. In the present study, dobutamine administration raised both CI and HR and lowered SVRI, which corroborates previous reports.

Colforsin activates adenylate cyclase in cardiomyocytes and vascular smooth muscle without mediating catecholamine beta-adrenoceptors. It increases cardiac contractility and reduces peripheral vascular resistance [11]. It was reported that colforsin improved cardiac contractility in isolated and acid-perfused rat heart under acidosis, and the cAMP level in cardiac muscle cells was higher in response to colforsin than to adrenaline [5]. Adrenaline binds not only to the catecholamine beta receptor but also to the alpha receptor so that its use *in vivo* can provide vasoconstrictor action. It is difficult to compare it with colforsin in the assessment of systemic and pulmonary vascular resistance. Therefore, we used dobutamine as a positive control in the present study because action of dobutamine, a beta-receptor agonist, is more appropriate to compare with the action of colforsin, and it is also widely used in the clinical situation. At clinical doses, dobutamine induced dose-related inotropism and afterload reduction with a relative lack of chronotropism. These conditions are appropriate for the management of patients with congestive heart failure. They could also improve renal blood flow by enhancing cardiac output and beta-2 adrenoceptor-stimulated vasodilation [10,12,13]. The colforsin doses administered in the present study (0.3 μg/kg/min, 0.6 μg/kg/min, and 1.2 μg/kg/min) were those required to increase the heart rate to a level equivalent to that induced by dobutamine in our preliminary study (S3 File). In the present study, colforsin administration increased CI and HR and decreased SVRI as did dobutamine. Therefore, colforsin could substitute for dobutamine as an inodilator and might be useful for the treatment of pathological conditions such as congestive heart failure.

In the present study, the acute respiratory acidosis canine model was induced by carbon dioxide inhalation [14]. The baseline pH at the time of dobutamine and colforsin administration was ~7.0. However, PaCO_2_ slightly increased during the experiment (0.5 h for stabilization and 3 h for measurement). Although the pH had slightly decreased by the end of the experiment, we believe that acute respiratory acidosis was induced in the dogs at ~pH 7.0.

Acute respiratory acidosis increases cardiac output and heart rate in dogs [15]. Symptoms of early hypercapnia include nausea/vomiting, muscle twitching, extrasystoles, and sympathetic nervous system stimulation. In the present study, the plasma adrenaline and noradrenaline levels in the Acidosis condition were significantly higher than those in the Normal condition. Therefore, elevated catecholamines could increase cardiac output and stroke volume under acidosis. Hypercapnia also causes anesthesia and peripheral blood vessel dilation [15]. At baseline, hypercapnia might have decreased systemic vascular resistance under acidosis in the present study.

The affinity of catecholamine for the beta-adrenoceptor decreases under acidosis [16]. In addition, in the present results, the cardiac output showed an interaction between both drugs and the acidosis condition and their cardiovascular effects were attenuated. Hypercapnia is anesthetic and suppresses cardiovascular function [17,18]. Consequently, even if the same isoflurane dose was administered to both groups, anesthesia may have been more profound in the Acidosis group than in the Normal group because the former presented with hypercapnia. For this reason, the effects of colforsin and dobutamine may have been further hampered by excessive anesthesia in hypercapnic condition.

Unlike dobutamine, the effect of colforsin on mPAP might be weak. Vascular smooth muscle in the pulmonary artery was relaxed by beta-adrenoceptor stimulation [19]. In terms of inodilator dose-response effects in rats, dobutamine increased systolic pulmonary artery pressure [20]. It also slightly elevated pulmonary vascular resistance in the anesthetized dog [21]. Pulmonary hypertension is defined as systolic PAP > 30 mm Hg or mPAP > 20 mm Hg [22]. In the present study, both colforsin and dobutamine increased the mPAP in a dose-dependent manner. Dobutamine at 10 μg/kg/min elevated the mPAP > 20 mm Hg in 2/6 dogs while 20 μg/kg/min dobutamine had the same effect on 3/6 dogs. In contrast, colforsin administration produced no pulmonary hypertension in Normal condition. In addition, all dogs administered with dobutamine showed pulmonary hypertension > 20 mm Hg of mPAP. Although certain dogs presented with pulmonary hypertension at high colforsin doses, their PAP was low relative to that induced by dobutamine. Even under acidosis, the influence of colforsin on pulmonary artery pressure was small compared with that of dobutamine. Left-sided heart disease is the most common cause of pulmonary hypertension in humans and dogs [22,23]. In a study of 60 dogs with pulmonary hypertension, 38 (63%) presented with degenerative mitral valve disease [24]. Other studies indicated that 14–31% of all dogs diagnosed with the latter disorder developed pulmonary hypertension [25,26]. Therefore, colforsin might be more efficacious than dobutamine in the treatment of severe mitral valve insufficiency accompanied by pulmonary hypertension. The effects of colforsin and dobutamine on the vascular smooth muscle of the pulmonary artery merit further investigation.

In the present study, plasma glucose level was higher in the Acidosis condition than in the Normal condition at baseline. Catecholamines markedly increase plasma glucose levels [27,28]. Insulin secretion declines after alpha adrenoceptor activation but rises in response to beta-2 adrenoceptor activation [29]. The high baseline plasma glucose level in the Acidosis condition was positively correlated with high plasma adrenaline and noradrenaline levels. Although dobutamine stimulates beta-1, beta-2, and alpha-1 adrenoceptors [7], the dobutamine dosage administered in this present study did not affect plasma glucose level under the Normal condition. The effects of alpha-1 catecholamine may have been offset by the beta-2 catecholaminic action of dobutamine. The effects of colforsin on insulin secretion are unknown. Nevertheless, the colforsin dose administered in the present study did not affect the plasma glucose level. Therefore, colforsin might be appropriate for diabetic patients whose cardiovascular function must be improved without raising their plasma glucose levels. In the future, the influence of colforsin administration on plasma insulin concentration should be investigated.

In the Acidosis condition, the baseline plasma potassium level was higher than that at the end of the experiment. Lactic acidosis is probably not associated with major intracellular shifts in potassium level. However, respiratory acidosis may influence serum potassium concentration [30]. In the Normal condition, neither dobutamine nor colforsin increased plasma potassium levels. Moreover, there was no significant difference in plasma potassium between the Normal and Acidosis conditions at baseline. Relative to the baseline, however, plasma potassium level was significantly higher in the Acidosis condition at the end of the experiment. Plasma potassium level rose in 3.5 h (0.5 h for stabilization and 3 h for the experiment) after the induction of acute respiratory acidosis. One acidotic dog receiving dobutamine (potassium level = 7.5 mmol/L) showed atrial stasis at the end of the experiment. Since the sample size was small in this assay, we could not confirm the relationship between dobutamine and arrhythmia. On the other hand, no arrhythmia was observed in dogs receiving colforsin (maximum potassium level = 8.3 mmol/L). Although there was no atrial stasis, colforsin acted as an inodilator here. Colforsin also suppressed digitalis- and epinephrine-induced ventricular arrhythmia models in dogs [31]. Abnormal plasma potassium levels and arrhythmia are often observed in the heart- and renal failure [32]. Unfortunately, the effect of both drugs on potassium could not be accurately evaluated because we had not utilized a true control group with placebo. In addition, calcium homeostasis is severely altered by acidosis and affects cardiac contractility. We did not measure a free/bound calcium in this study. In the future, the associations among colforsin, plasma potassium and calcium level, and arrhythmia in these diseases should be investigated.

Some dogs presented with transient nausea or vomiting during recovery from both drugs. Dobutamine has provoked nausea, headache, vomiting, and dyspnea. To the best of our knowledge, adverse effects have not been reported for colforsin. Nevertheless, it still may have side effects similar to those of dobutamine. The present study aimed to investigate the cardiovascular effects of colforsin under acidosis. Since the dose administered was impractical, many side effects may have been induced. In future research, we could endeavor to optimize the dosage of colforsin, which would improve cardiovascular function in the presence of respiratory- or another type of acidosis. Certain dogs under the Acidosis condition showed miosis at the end of the experiment. Miosis occurs when intracranial pressure increases. Since hypercapnia increases cerebral blood flow [33], it may have also elevated intracranial pressure and induced miosis. Dogs presenting with miosis returned to normal pupillary diameter within 6 h after the experiment. No other neurological complications were observed. Although acute respiratory acidosis was maintained for 3.5 h in the present study, pupil size should be verified in respiratory acidosis and permissive hypercapnia in a clinical setting.

We conducted this study assuming that pulmonary edema may complicate respiratory acidosis. However, there were certain limitations here. Although oxygenation is impaired in pulmonary edema, we did not conduct this experiment under hypoxemia, which stimulates the sympathetic nervous system and enhances cardiovascular function. We wanted to clarify the cardiovascular effects of colforsin in normal dogs. Therefore, we conducted this experiment with 100% oxygen carrier gas. Next, we used 3 female and 3 male healthy dogs free of heart or lung disease. Although it has been reported that there is a gender difference in the effects of catecholamine [34], it was not possible to clarify the effect of colforsin with respect to genders due to the small number of samples in this present study. In addition, dogs with severe mitral valve insufficiency causing pulmonary edema already has depressed cardiorespiratory function. Therefore, administering colforsin and dobutamine to these patients may produce different cardiovascular effects. Further studies are needed to establish the effects of colforsin and dobutamine on gender and these disease models and clinical cases and to verify the safety and efficacy of colforsin. In turn, these findings could be adapted to human medicine.

## Conclusions

The cardiovascular effects of colforsin and dobutamine are similar in healthy beagles under isoflurane anesthesia. In acute respiratory acidosis induced by carbon dioxide inhalation, the cardiovascular function was enhanced by endogenous catecholamine secretion. In addition, the rates of change in CI, HR, and SVRI caused by colforsin and dobutamine administration were attenuated. Therefore, it may be necessary to increase the colforsin and dobutamine doses under respiratory acidosis relative to those administered under the normal condition. Since colforsin had little effect on the mPAP, it may be more suitable as an inodilator than dobutamine in the treatment of diseases which increase the mPAP. Our next steps are to induce a pulmonary hypertension canine model, confirm the effects of colforsin on it, adapt colforsin administration for patients with pulmonary hypertension in our institution, and compare its efficacy with that of existing catecholamines.

## Supporting information

S1 File Blood gas and biochemical measurements apparatus information. (PDF)

S2 File Linear mixed-effects model results for the effects of colforsin/dobutamine and pH condition on cardiovascular parameters. (PDF)

S3 File Effect of colforsin and dobutamine on heart rate during Normal condition in three dogs under isoflurane anesthesia: Preliminary study. (PDF)

S1 Table The effects of colforsin on blood gas examination and blood biochemical test in six anesthetized dogs in eucapnia (Normal) and acute respiratory acidosis (Acidosis) at baseline and at the end of the experiment. (PDF)

S2 Table Descriptive statistics value of cardiovascular parameters. (PDF)
