# Supplementary material for: Cardiovascular effects of intravenous colforsin in normal and acute respiratory acidosis canine models: A dose-response study
Source: PLoS One. 2019 Jul 10;14(7):e0213414. doi: 10.1371/journal.pone.0213414 (PMC6619603; doi:10.1371/journal.pone.0213414)
Supplement: S1 File — (PDF) [file pone.0213414.s001.pdf]

Blood gas measurements apparatus: GEM-Premier 3000; IL Japan Co. Ltd., Tokyo, Japan.

- **Sample:** 2mL of whole blood with lithium heparin from arterial catheter
- **Measurement time:** Immediately

Parameters	Measurement principle	Coefficient of Variation	r^2
pH	Potentiometry	≦1%	>0.99 (6.80-8.00)
$PaCO_2$ (mmHg)	Potentiometry	≦4%	>0.99 (15-125)
PaO_2 (mmHg)	Amperometry	≦5%	>0.99 (30-600)
Lactate (mmol/L)	Amperometry	≦5%	>0.99 (0.3-17)
PCV (%)	Conductimetry	≦2%	>0.99 (18-65)
HCO_3^- (mEq/L)	Calculated value	not listed	not listed
BEecf (mEq/L)	Calculated value	not listed	not listed

$PaCO_2$, arterial partial pressure of carbon dioxide; PaO_2, arterial partial pressure of oxygen; HCO_3^-, bicarbonate ion; BEecf, base excess in the extracellular fluid; PCV, packed cell volume

Biochemical measurements apparatus: DRI-CHEM 7000V; Fujifilm Co. Ltd., Tokyo, Japan

- **Sample:** 2mL of whole blood with heparin from arterial catheter
- **Preprocessing:** Immediately centrifuged (1,000 × g for 10 min at 4° C) to separate the plasma
- **Measurement time:** Immediately

Parameters	Measurement principle	Coefficient of Variation	r^2
Glucose (mg/dL)	Enzymatic method	≦5%	>0.99 (10-600)
BUN (mg/dL)	Enzymatic method	≦6%	>0.99 (5-140)
Creatinine (mg/dL)	Enzymatic method	≦5%	>0.99 (0.2-24)
Na (mEq/L)	Ion selective electrode method	not listed	>0.99 (75-250)
K (mEq/L)	Ion selective electrode method	not listed	>0.99 (1-14)
Cl (mEq/L)	Ion selective electrode method	not listed	>0.99 (50-175)

BUN, blood urea nitrogen; Na, sodium ion; K, potassium ion, Cl, chloride ion

Catecholamine measurements: External commercial laboratory (BML Inc., Tokyo, Japan) which is certified to the international standard International Organization for Standardization (ISO) 15189 and accredited the College of American Pathologists (CAP) by the United States.

- **Sample:** 2mL of whole blood with ethylene diamine tetra acetic acid from arterial catheter
- **Preprocessing:** Immediately centrifuged (1,000 × g for 10 min at 4° C) to separate the plasma
- **Store:** Frozen at -80 °C one night and thawed once just before the measurement

Parameters	Measurement principle	Coefficient of Variation
Adrenaline (ng/mL)	HPLC	not listed (Guaranteed quality)
Noradrenaline (ng/mL)	HPLC	not listed (Guaranteed quality)
Dopamine (ng/mL)	HPLC	not listed (Guaranteed quality)

HPLC, high performance liquid chromatography
