# Supplementary material for: Cardiovascular effects of intravenous colforsin in normal and acute respiratory acidosis canine models: A dose-response study
Source: PLoS One. 2019 Jul 10;14(7):e0213414. doi: 10.1371/journal.pone.0213414 (PMC6619603; doi:10.1371/journal.pone.0213414)
Supplement: S2 File — (PDF) [file pone.0213414.s002.pdf]

Linear mixed-effects model results for the effects of colforsin/dobutamine and pH condition on heart rate.

Variables	Estimate	Standard error	*P*-value
Colforsin			
Intercept	80.6	5.7	<0.001
Fixed effects			
Dose of colforsin	94.5	3.6	<0.001
Acidosis (normal as reference)	32.2	3.6	<0.001
Dose: acidosis (normal as reference)	-69.7	5.1	<0.001
Random effect	Variance	Variance of residual	ICC%*
Dogs	160.6	226.3	41.5
Variables	Estimate	Standard error	*P*-value
Dobutamine			
Intercept	91.9	9.8	<0.001
Fixed effects			
Dose of dobutamine	4.9	0.3	<0.001
Acidosis (normal as reference)	23.6	4.1	<0.001
Dose: acidosis (normal as reference)	-2.4	0.4	<0.001
Random effect	Variance	Variance in residual	ICC%*
Dogs	528.7	258.4	67.1

*ICC: Intra class correlation percentage: variance of random effect / total variance

Linear mixed-effects model results for the effects of colforsin/dobutamine and pH condition on heart rate.

Variables	Estimate	Standard error	*P*-value
Colforsin			
Intercept	46.4	2.1	<0.001
Fixed effects			
Dose of colforsin	0.9	1.4	0.510
Acidosis (normal as reference)	6.3	1.4	<0.001
Dose: acidosis (normal as reference)	4.4	2.0	0.034
Random effect	Variance	Variance of residual	ICC%*
Dogs	20.5	35.4	36.7
Variables	Estimate	Standard error	*P*-value
Dobutamine			
Intercept	47.8	2.2	<0.001
Fixed effects			
Dose of dobutamine	0.5	0.1	<0.001
Acidosis (normal as reference)	7.5	1.6	<0.001
Dose: acidosis (normal as reference)	0.1	0.1	0.610
Random effect	Variance	Variance in residual	ICC%*
Dogs	21.9	37.8	36.7

*ICC: Intra class correlation percentage: variance of random effect / total variance

Linear mixed-effects model results for the effects of colforsin/dobutamine and pH condition on systolic artery pressure.

Variables	Estimate	Standard error	*P*-value
Colforsin			
Intercept	100.3	3.4	<0.001
Fixed effects			
Dose of colforsin	-15.0	2.6	<0.001
Acidosis (normal as reference)	-6.3	2.6	0.016
Dose: acidosis (normal as reference)	1.1	3.7	0.764
Random effect	Variance	Variance of residual	ICC%*
Dogs	51.3	116.7	30.5
Variables	Estimate	Standard error	*P*-value
Dobutamine			
Intercept	97.0	3.7	<0.001
Fixed effects			
Dose of dobutamine	-0.8	0.2	<0.001
Acidosis (normal as reference)	2.3	3.1	0.459
Dose: acidosis (normal as reference)	-0.5	0.3	0.081
Random effect	Variance	Variance in residual	ICC%*
Dogs	1.44	1.03	58.3

*ICC: Intra class correlation percentage: variance of random effect / total variance

Linear mixed-effects model results for the effects of colforsin/dobutamine and pH condition on mean artery pressure.

Variables	Estimate	Standard error	*P*-value
Colforsin			
Intercept	72.3	3.2	<0.001
Fixed effects			
Dose of colforsin	-12.3	1.9	<0.001
Acidosis (normal as reference)	-5.0	1.9	0.010
Dose: acidosis (normal as reference)	3.4	2.7	0.216
Random effect	Variance	Variance of residual	ICC%*
Dogs	51.0	64.8	44.1
Variables	Estimate	Standard error	*P*-value
Dobutamine			
Intercept	69.2	2.1	<0.001
Fixed effects			
Dose of dobutamine	-0.5	0.1	0.001
Acidosis (normal as reference)	2.5	2.3	0.260
Dose: acidosis (normal as reference)	-0.4	0.2	0.053
Random effect	Variance	Variance in residual	ICC%*
Dogs	11.3	77.2	12.8

*ICC: Intra class correlation percentage: variance of random effect / total variance

Linear mixed-effects model results for the effects of colforsin/dobutamine and pH condition on diastolic artery pressure.

Variables	Estimate	Standard error	*P*-value
Colforsin			
Intercept	56.6	2.4	<0.001
Fixed effects			
Dose of colforsin	-4.2	1.5	0.006
Acidosis (normal as reference)	-6.7	1.5	<0.001
Dose: acidosis (normal as reference)	-3.0	2.2	0.172
Random effect	Variance	Variance of residual	ICC%*
Dogs	29.4	39.6	42.6
Variables	Estimate	Standard error	*P*-value
Dobutamine			
Intercept	54.7	1.7	<0.001
Fixed effects			
Dose of dobutamine	-0.6	0.1	<0.001
Acidosis (normal as reference)	0.1	1.8	0.974
Dose: acidosis (normal as reference)	-0.1	0.2	0.367
Random effect	Variance	Variance in residual	ICC%*
Dogs	7.3	46.5	13.6

*ICC: Intra class correlation percentage: variance of random effect / total variance

Linear mixed-effects model results for the effects of colforsin/dobutamine and pH condition on right atrial pressure.

Variables	Estimate	Standard error	*P*-value
Colforsin			
Intercept	3.7	0.3	<0.001
Fixed effects			
Dose of colforsin	-1.1	0.2	<0.001
Acidosis (normal as reference)	0.8	0.2	<0.001
Dose: acidosis (normal as reference)	0.5	0.3	0.091
Random effect	Variance	Variance of residual	ICC%*
Dogs	0.38	0.86	30.6
Variables	Estimate	Standard error	*P*-value
Dobutamine			
Intercept	3.07	0.53	<0.001
Fixed effects			
Dose of dobutamine	-0.03	0.01	0.028
Acidosis (normal as reference)	1.77	0.19	<0.001
Dose: acidosis (normal as reference)	0.01	0.02	0.574
Random effect	Variance	Variance in residual	ICC%*
Dogs	1.58	0.57	73.5

*ICC: Intra class correlation percentage: variance of random effect / total variance

Linear mixed-effects model results for the effects of colforsin/dobutamine and pH condition on systemic vascular resistance index.

Variables	Estimate	Standard error	*P*-value
Colforsin			
Intercept	5,827.6	248.8	<0.001
Fixed effects			
Dose of colforsin	-3,356.4	177.9	<0.001
Acidosis (normal as reference)	-2,448.7	178.4	<0.001
Dose: acidosis (normal as reference)	2,148.9	254.2	<0.001
Random effect	Variance	Variance of residual	ICC%*
Dogs	280,288.8	553,048.8	33.6
Variables	Estimate	Standard error	*P*-value
Dobutamine			
Intercept	5,617.7	480.6	<0.001
Fixed effects			
Dose of dobutamine	-203.2	17.5	<0.001
Acidosis (normal as reference)	-2,290.2	282.8	<0.001
Dose: acidosis (normal as reference)	97.5	24.4	<0.001
Random effect	Variance	Variance in residual	ICC%*
Dogs	11,432,245.0	1,212,291.3	48.5

*ICC: Intra class correlation percentage: variance of random effect / total variance

Linear mixed-effects model results for the effects of colforsin/dobutamine and pH condition on pulmonary artery occlusion pressure.

Variables	Estimate	Standard error	*P*-value
Colforsin			
Intercept	4.4	0.4	<0.001
Fixed effects			
Dose of colforsin	-1.1	0.3	<0.001
Acidosis (normal as reference)	6.1	0.3	<0.001
Dose: acidosis (normal as reference)	-0.9	0.4	0.018
Random effect	Variance	Variance of residual	ICC%*
Dogs	0.6	1.1	36.5
Variables	Estimate	Standard error	*P*-value
Dobutamine			
Intercept	4.80	0.60	<0.001
Fixed effects			
Dose of dobutamine	-0.02	0.01	0.173
Acidosis (normal as reference)	5.33	0.23	<0.001
Dose: acidosis (normal as reference)	0.02	0.02	0.425
Random effect	Variance	Variance in residual	ICC%*
Dogs	1.99	0.84	70.3

*ICC: Intra class correlation percentage: variance of random effect / total variance

Linear mixed-effects model results for the effects of colforsin/dobutamine and pH condition on mean pulmonary artery pressure.

Variables	Estimate	Standard error	*P*-value
Colforsin			
Intercept	10.39	0.69	<0.001
Fixed effects			
Dose of colforsin	2.48	0.38	<0.001
Acidosis (normal as reference)	7.19	0.38	<0.001
Dose: acidosis (normal as reference)	-0.03	0.55	0.9598
Random effect	Variance	Variance of residual	ICC%*
Dogs	2.42	2.56	48.6
Variables	Estimate	Standard error	*P*-value
Dobutamine			
Intercept	12.23	0.71	<0.001
Fixed effects			
Dose of dobutamine	0.42	0.03	<0.001
Acidosis (normal as reference)	8.10	0.44	<0.001
Dose: acidosis (normal as reference)	-0.06	0.04	0.120
Random effect	Variance	Variance in residual	ICC%*
Dogs	2.38	3.04	43.9

*ICC: Intra class correlation percentage: variance of random effect / total variance

Linear mixed-effects model results for the effects of colforsin/dobutamine and pH condition on pulmonary vascular resistance index.

Variables	Estimate	Standard error	*P*-value
Colforsin			
Intercept	561.8	55.2	<0.001
Fixed effects			
Dose of colforsin	-167.7	21.4	<0.001
Acidosis (normal as reference)	-132.5	21.5	<0.001
Dose: acidosis (normal as reference)	196.8	30.6	<0.001
Random effect	Variance	Variance of residual	ICC%*
Dogs	16964.3	8035.9	67.9
Variables	Estimate	Standard error	*P*-value
Dobutamine			
Intercept	608.4	60.7	<0.001
Fixed effects			
Dose of dobutamine	-3.6	1.4	0.009
Acidosis (normal as reference)	-100.5	22.1	<0.001
Dose: acidosis (normal as reference)	3.6	1.9	0.063
Random effect	Variance	Variance in residual	ICC%*
Dogs	20589.9	7515.1	73.3

*ICC: Intra class correlation percentage: variance of random effect / total variance
