# Supplementary material for: Cardiovascular effects of intravenous colforsin in normal and acute respiratory acidosis canine models: A dose-response study
Source: PLoS One. 2019 Jul 10;14(7):e0213414. doi: 10.1371/journal.pone.0213414 (PMC6619603; doi:10.1371/journal.pone.0213414)
Supplement: S3 File — (PDF) [file pone.0213414.s003.pdf]

Effect of colforsin and dobutamine on heart rate during Normal condition in three dogs under isoflurane anesthesia: preliminary study.

| **Colforsin** | **0μg/kg/min** | | | **0.15μg/kg/min** | | | **0.3μg/kg/min** | | | **0.6μg/kg/min** | | | **1.2μg/kg/min** | | | **2.4μg/kg/min** | | |
|---|---|---|---|---|---|---|---|---|---|---|---|---|---|---|---|---|---|---|
| **Dog No.** | **20m** | **40m** | **60m** | **20m** | **40m** | **60m** | **20m** | **40m** | **60m** | **20m** | **40m** | **60m** | **20m** | **40m** | **60m** | **20m** | **40m** | **60m** |
| **A** | **88** | **89** | **92** | **92** | **94** | **95** | **92** | **96** | **102** | **105** | **120** | **147** | **165** | **176** | **182** | **193** | **195** | **196** |
| **B** | **72** | **76** | **75** | **83** | **83** | **84** | **85** | **89** | **96** | **101** | **133** | **152** | **173** | **182** | **191** | **197** | **205** | **204** |
| **C** | **100** | **101** | **97** | **102** | **105** | **105** | **103** | **107** | **115** | **117** | **145** | **166** | **180** | **193** | **201** | **205** | **201** | **210** |
| **Mean** | **86.7** | **88.7** | **88** | **92.3** | **94** | **94.7** | **93.3** | **97.3** | **104** | **108** | **133** | **155** | **173** | **184** | **191** | **198** | **200** | **203** |
| **StdDev** | **11.5** | **10.2** | **9.42** | **7.76** | **8.98** | **8.58** | **7.41** | **7.41** | **7.93** | **6.8** | **10.2** | **8.04** | **6.13** | **7.04** | **7.76** | **4.99** | **4.11** | **5.73** |

| **Dobutamine** | **0μg/kg/min** | | | **2.5μg/kg/min** | | | **5μg/kg/min** | | | **10μg/kg/min** | | | **20μg/kg/min** | | | **40μg/kg/min** | | |
|---|---|---|---|---|---|---|---|---|---|---|---|---|---|---|---|---|---|---|
| **Dog No.** | **20m** | **40m** | **60m** | **20m** | **40m** | **60m** | **20m** | **40m** | **60m** | **20m** | **40m** | **60m** | **20m** | **40m** | **60m** | **20m** | **40m** | **60m** |
| **A** | **95** | **93** | **94** | **93** | **100** | **94** | **96** | **102** | **105** | **118** | **145** | **148** | **185** | **188** | **185** | **195** | **198** | **204** |
| **B** | **83** | **81** | **81** | **88** | **82** | **80** | **92** | **104** | **100** | **112** | **138** | **141** | **190** | **187** | **191** | **205** | **211** | **208** |
| **C** | **102** | **98** | **105** | **92** | **94** | **90** | **95** | **117** | **120** | **132** | **163** | **158** | **188** | **205** | **201** | **213** | **221** | **214** |
| **Mean** | **93.3** | **90.7** | **93.3** | **91** | **92** | **88** | **94.3** | **108** | **108** | **121** | **149** | **149** | **188** | **193** | **192** | **204** | **210** | **209** |
| **StdDev** | **7.85** | **7.13** | **9.81** | **2.16** | **7.48** | **5.89** | **1.7** | **6.65** | **8.5** | **8.38** | **10.5** | **6.98** | **2.05** | **8.26** | **6.6** | **7.36** | **9.42** | **4.11** |

.

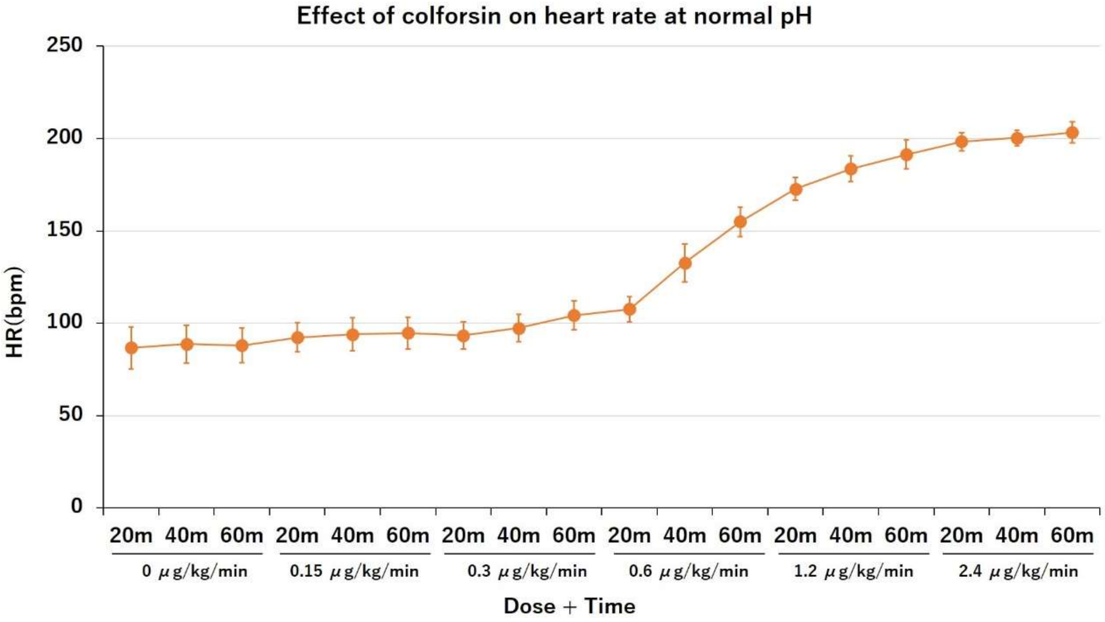


The effect of colforsin on heart rate at normal condition. The plot and error bar show mean value and standard deviation, respectively. The horizontal axis shows the dosage of colforsin (0, 0.15, 0.3, 0.6, 1.2, and 2.4 μg/kg/min) and time elapsed time.

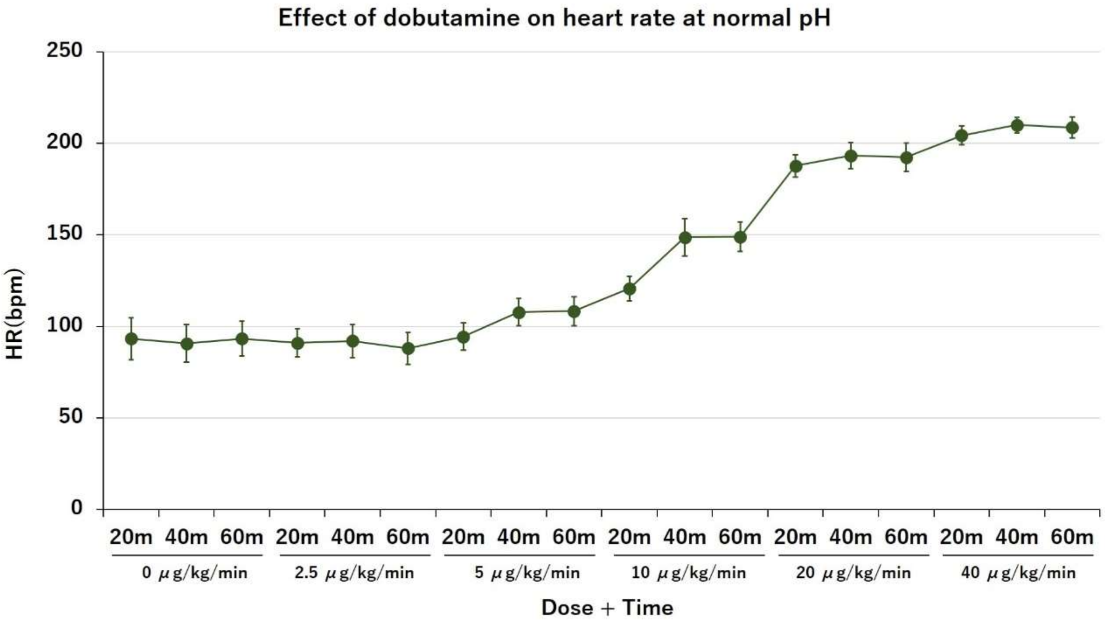


The effect of colforsin on heart rate at normal condition. The plot and error bar show mean value and standard deviation, respectively. The horizontal axis shows the dosage of colforsin (0, 2.5, 5, 10, 20, and 40 μg/kg/min) and time elapsed time.
