# Supplementary material for: Cardiovascular effects of intravenous colforsin in normal and acute respiratory acidosis canine models: A dose-response study
Source: PLoS One. 2019 Jul 10;14(7):e0213414. doi: 10.1371/journal.pone.0213414 (PMC6619603; doi:10.1371/journal.pone.0213414)
Supplement: S1 Table — (PDF) [file pone.0213414.s004.pdf]

The effects of colforsin on blood gas examination and blood biochemical test in six anesthetized dogs in eucapnia (Normal) and acute respiratory acidosis (Acidosis) at baseline and at the end of the experiment.

Variable (Unit)	Condition	Baseline	End of experiment	Reference
Adrenaline (ng/mL	Normal	0.01 [0.01–0.09]	–	<0.10
	Acidosis	0.14 [0.05–0.37]§	–	
Noradrenaline (ng/mL)	Normal	0.03 [0.01–0.46]	–	0.10~0.50
	Acidosis	0.35 [0.17–0.97]§	–	
Dopamine (ng/mL)	Normal	0.01 [0.01–0.04]	–	<0.03
	Acidosis	0.02 [0.02–0.08]	–	
PCV (%)	Normal	28 [24–37]	26 [21–34]	37–55
	Acidosis	39 [29–47]	35 [25–37]	
pH	Normal	7.38 [7.34–7.42]	7.32 [7.29–7.37]*	7.35–7.45
	Acidosis	7.04 [7.01–7.08]†	6.99 [6.92–7.01]‡	
$PaCO_2$ (mm Hg)	Normal	40 [34–41]	39 [35–45]	30.8–42.8
	Acidosis	98 [92–100]†	110 [106–125]‡	
PaO_2 (mm Hg)	Normal	559 [501–616]	571 [496–619]	80.9–103.3
	Acidosis	522 [488–538]	519 [387–540]	
HCO_3^- (mEq/L)	Normal	22.6 [20.8–25.4]	20.3 [19.6–23.2]	18.8–25.6
	Acidosis	26.1 [24.6–27.7]†	27.0 [23.8–28.0]	
DaO_2I (mL O_2/min/kg)	Normal	27.1 [17.9–32.3]	56.0 [42.3–75.0]*	–
	Acidosis	50.3 [37.2–62.6]†	61.2 [50.3–71.2]	
VaO_2I (mL O_2/min/kg)	Normal	3.9 [2.5–5.6]	6.5 [4.7–7.4]	–
	Acidosis	4.4 [3.1–5.0]	5.5 [2.8–6.3]	
O_2ER (%)	Normal	16.4 [12.4–20.0]	11.1 [8.1–12.7]	–
	Acidosis	8.3 [7.0–10.5]†	8.6 [5.5–10.7]	
BEecf (mEq/L)	Normal	-2.2 [-4.5–1.0]	-5.6 [-6.5–-3.0]	-4–+4
	Acidosis	-4.5 [-7.0–-2.8]	-4.6 [-8.0–-3.0]	
Lactate (mmol/L)	Normal	1.4 [1.1–2.2]	0.9 [0.7–1.2]*	<2.0
	Acidosis	0.5 [0.3–1.0]†	0.3 [0.3–0.5]	
Na (mEq/L)	Normal	144 [143–145]	145 [144–148]	135–147
	Acidosis	146 [142–150]	144 [139–148]	
K (mEq/L)	Normal	3.8 [3.2–3.9]	3.5 [3.2–3.8]	3.5–5.0
	Acidosis	3.9 [3.8–4.2]	7.2 [6.2–8.3]‡	
Cl (mEq/L)	Normal	115 [111–116]	117 [109–121]	95–125
	Acidosis	115 [110–118]	115 [113–119]	
Glucose (mg/dL)	Normal	104 [92–117]	108 [89–115]	60–110
	Acidosis	136 [114–188]†	128 [117–159]	
BUN (mg/dL)	Normal	13.2 [9.0–17.5]	13.8 [10.0–20.0]	10–20
	Acidosis	15.5 [12.6–22.0]	19.0 [15.6–25.0]	
Creatinine (mg/dL)	Normal	0.4 [0.3–0.6]	0.4 [0.2–0.5]	0.6–1.2
	Acidosis	0.6 [0.4–0.7]	0.9 [0.6–1.2]	

PCV, packed cell volume; $PaCO_2$, arterial partial pressure of carbon dioxide; PaO_2, arterial partial pressure of oxygen; HCO_3^-, bicarbonate ion; DaO_2^-, oxygen delivery; VaO_2^-, oxygen consumption; O_2ER, oxygen extraction ratio; BEecf, base excess in the extracellular fluid; Na, sodium ion; K, potassium ion, Cl, chloride ion; BUN, blood urea nitrogen. The reference values were shown from individual testing apparatus. § shows significant difference ($P < 0.05$) from baseline in Normal condition by Wilcoxson signed-rank test. * and † show significant difference ($P < 0.05$) from baseline in Normal condition, respectively, and ‡ shows significant difference ($P < 0.05$) from baseline in Acidosis condition by Steel-Dwass, respectively.

The effects of dobutamine on blood gas examination and blood biochemical test in six anesthetized dogs in eucapnia (Normal) and acute respiratory acidosis (Acidosis) at baseline and at the end of the experiment.

Variable (Unit)	Condition	Baseline	End of experiment	Reference
Adrenaline (ng/mL)	Normal	0.01 [0.01–0.13]	–	<0.10
	Acidosis	0.26 [0.08–2.08]§	–	
Noradrenaline (ng/mL)	Normal	0.04 [0.02–0.09]	–	0.10–0.50
	Acidosis	0.32 [0.24–0.44]§	–	
Dopamine (ng/mL)	Normal	0.01 [0.01–0.02]	–	<0.03
	Acidosis	0.02 [0.01–0.03]	–	
PCV (%)	Normal	34 [27–39]	35 [33–43]	37–55
	Acidosis	40 [34–49]	45 [40–51]	
pH	Normal	7.38 [7.33–7.41]	7.30 [7.25–7.36]*	7.35–7.45
	Acidosis	6.99 [6.96–7.05]†	6.92 [6.86–6.95]‡	
$PaCO_2$ (mm Hg)	Normal	38 [36–42]	42 [35–46]	30.8~42.8
	Acidosis	109 [101–114]†	126 [115–146]‡	
PaO_2 (mm Hg)	Normal	539 [495–571]	579 [568–607]*	80.9–103.3
	Acidosis	525 [443–551]	505 [473–544]	
HCO_3^- (mEq/L)	Normal	22.8 [21.9–24.6]	20.1 [18–21.8]*	18.8–25.6
	Acidosis	26.6 [24.6–27.9]†	25.9 [24.7–27.9]	
DaO_2I (mL O_2/min/kg)	Normal	31.7 [20.6–40.7]	89.9 [67.4–109.6]*	–
	Acidosis	49.1 [44.5–59.7]†	101.1 [83.3–112.4]‡	
VaO_2I (mL O_2/min/kg)	Normal	4.0 [2.4–4.3]	7.0 [5.5–7.8]*	–
	Acidosis	4.4 [3.3–6.2]	6.5 [4.2–7.3]	
O_2ER (%)	Normal	13.5 [11.2–17.0]	7.8 [6.1–9.5]*	–
	Acidosis	8.4 [7.1–11.9]	6.1 [4.6–7.9]	
BEecf (mEq/L)	Normal	-2.0 [-3.2–-1.0]	-7.0 [-8.1–-4.0]	-4–+4
	Acidosis	-5.0 [-7.0–-2.6]	-6.6 [-8.0–-5.2]	
Lactate (mmol/L)	Normal	1.5 [0.6–3.2]	0.3 [0.3–0.5]*	<2.0
	Acidosis	0.6 [0.3–0.9]	0.6 [0.3–1.6]	
Na (mEq/L)	Normal	144 [142–148]	146 [143–148]	135–147
	Acidosis	146 [144–148]	146 [141–148]	
K (mEq/L)	Normal	3.7 [3.3–4.3]	3.5 [3.0–4.8]	3.5–5.0
	Acidosis	3.8 [3.2–4.3]	6.1 [5.0–7.5]‡	
Cl (mEq/L)	Normal	115 [111–119]	116 [115–123]	95–125
	Acidosis	113 [109–118]	113 [111–117]	
Glucose (mg/dL)	Normal	103 [89–137]	94 [87–106]	60–110
	Acidosis	150 [123–200]†	138 [121–243]	
BUN (mg/dL)	Normal	15.0 [12.1–17.4]	13.0 [11.0–15.7]	10–20
	Acidosis	15.0 [11.0–22.0]	18.5 [15.0–25.0]	
Creatinine (mg/dL)	Normal	0.5 [0.4–0.9]	0.35 [0.3–0.5]	0.6–1.2
	Acidosis	0.6 [0.5–0.7]	1.05 [0.6–1.4]	

PCV, packed cell volume; $PaCO_2$, arterial partial pressure of carbon dioxide; PaO_2, arterial partial pressure of oxygen; HCO_3^-, bicarbonate ion; DaO_2I, oxygen delivery; VaO_2I, oxygen consumption; O_2ER, oxygen extraction ratio; BEecf, base excess in the extracellular fluid; Na, sodium ion; K, potassium ion, Cl, chloride ion; BUN, blood urea nitrogen. The reference values were shown from individual testing apparatus. § shows significant difference ($P < 0.05$) from baseline in Normal condition by Wilcoxson signed-rank test. * and † show significant difference ($P < 0.05$) from baseline in Normal condition, respectively, and ‡ shows significant difference ($P < 0.05$) from baseline in Acidosis condition by Steel-Dwass, respectively.
