# Supplementary material for: Cardiovascular effects of intravenous colforsin in normal and acute respiratory acidosis canine models: A dose-response study
Source: PLoS One. 2019 Jul 10;14(7):e0213414. doi: 10.1371/journal.pone.0213414 (PMC6619603; doi:10.1371/journal.pone.0213414)
Supplement: S2 Table — (PDF) [file pone.0213414.s005.pdf]

Descriptive statistics value: The effect of colforsin on cardiac index (L/min/m^2)

	Colforsin at Normal condition				Colforsin at Acidosis condition			
Variables	baseline	1st. Dose	2nd. Dose	3rd. Dose	baseline	1se. Dose	2nd. Dose	3rd. Dose
N	21	23	26	23	21	23	26	23
Sum total	81.27	118.69	181.58	215.87	81.27	118.69	181.58	215.87
Mean	3.87	5.16	6.98	9.39	3.87	5.16	6.98	9.39
Standard deviation	0.89	1.68	1.91	1.19	0.89	1.68	1.91	1.19
Standard error	0.19	0.35	0.37	0.25	0.19	0.35	0.37	0.25
Range	2.80	4.38	6.14	3.70	2.80	4.38	6.14	3.70
Minimum	2.33	3.53	4.43	7.70	2.33	3.53	4.43	7.70
Maximum	5.13	7.91	10.57	11.39	5.13	7.91	10.57	11.39
Median	4.20	4.52	6.08	9.11	4.20	4.52	6.08	9.11
25% quartile	3.04	3.84	5.71	8.30	3.04	3.84	5.71	8.30
75% quartile	4.39	7.36	9.11	10.43	4.39	7.36	9.11	10.43
Interval estimation of population mean	Reliability	95%			Reliability	95%		
Lower limit (t-distribution)	3.46	4.43	6.21	8.87	3.46	4.43	6.21	8.87
Upper limit (t-distribution)	4.28	5.89	7.75	9.90	4.28	5.89	7.75	9.90

Descriptive statistics value: The effect of dobutamine on cardiac index (L/min/m^2)

	Dobutamine at Normal condition				Dobutamine at Acidosis condition			
Variables	baseline	1st. Dose	2nd. Dose	3rd. Dose	baseline	1se. Dose	2nd. Dose	3rd. Dose
N	18	18	18	18	19	19	18	20
Sum total	73.16	99.83	156.02	179.47	106.37	151.56	167.29	202.73
Mean	4.06	5.55	8.67	9.97	5.60	7.98	9.29	10.14
Standard deviation	1.21	1.89	1.26	0.78	0.93	1.68	1.32	1.44
Standard error	0.28	0.44	0.30	0.18	0.21	0.39	0.31	0.32
Range	3.57	5.12	3.77	2.62	2.64	4.59	4.23	5.10
Minimum	2.41	2.76	6.57	8.74	4.23	5.67	7.02	7.77
Maximum	5.98	7.88	10.33	11.37	6.87	10.25	11.25	12.87
Median	3.95	6.00	9.02	9.77	5.51	7.72	9.30	10.13
25% quartile	3.05	3.60	7.60	9.42	4.76	6.55	8.69	9.57
75% quartile	5.16	7.34	9.58	10.79	6.46	9.34	10.16	10.50
Interval estimation of population mean	Reliability	95%			Reliability	95%		
Lower limit (t-distribution)	3.46	4.61	8.04	9.58	5.15	7.17	8.64	9.46
Upper limit (t-distribution)	4.66	6.48	9.30	10.36	6.04	8.79	9.95	10.81

Descriptive statistics value: The effect of colforsin on heart rate (beats/min)

	Colforsin at Normal condition				Colforsin at Acidosis condition			
Variables	baseline	1st. Dose	2nd. Dose	3rd. Dose	baseline	1se. Dose	2nd. Dose	3rd. Dose
N	21	23	26	23	19	21	22	23
Sum total	1807.00	2320.00	3521.00	4517.00	2194.00	2407.00	2828.00	3271.00
Mean	86.05	100.87	135.42	196.39	115.47	114.62	128.55	142.22
Standard deviation	17.91	22.18	31.43	7.21	14.16	13.42	15.59	15.15
Standard error	3.91	4.62	6.16	1.50	3.25	2.93	3.32	3.16
Range	49	66	80	26	43	34	53	51
Minimum	67	75	94	184	92	98	103	114
Maximum	116	141	174	210	135	132	156	165
Median	80	88	150	196	115	118	124	137
25% quartile	74	82	105	192	106	99	117	132
75% quartile	109	115	165.5	199	126	128	140.25	158
Interval estimation of population mean	Reliability	95%			Reliability	95%		
Lower limit (t-distribution)	77.90	91.28	122.73	193.27	108.65	108.51	121.63	135.66
Upper limit (t-distribution)	94.20	110.46	148.12	199.51	122.30	120.73	135.46	148.77

Descriptive statistics value: The effect of dobutamine on heart rate (beats/min)

	Dobutamine at Normal condition				Dobutamine at Acidosis condition			
Variables	baseline	1st. Dose	2nd. Dose	3rd. Dose	baseline	1se. Dose	2nd. Dose	3rd. Dose
N	18	18	18	18	19	19	18	20
Sum total	1712.00	1852.00	2788.00	3358.00	2092.00	2508.00	2645.00	3212.00
Mean	95.11	102.89	154.89	186.56	110.11	132.00	146.94	160.60
Standard deviation	25.52	33.42	34.76	11.42	18.81	30.70	23.24	20.21
Standard error	6.01	7.88	8.19	2.69	4.31	7.04	5.48	4.52
Range	78	85	96	37	56	86	62	58
Minimum	60	58	109	164	82	89	120	134
Maximum	138	143	205	201	138	175	182	192
Median	96	103	159.5	188.5	110	118	142	160
25% quartile	73.25	67	116.5	181.25	92.5	107.5	124.75	139.75
75% quartile	108.75	141.5	179.75	194	130.5	157	173	180.25
Interval estimation of population mean	Reliability	95%			Reliability	95%		
Lower limit (t-distribution)	82.42	86.27	137.60	180.87	101.04	117.20	135.39	151.14
Upper limit (t-distribution)	107.80	119.51	172.18	192.24	119.17	146.80	158.50	170.06

Descriptive statistics value: The effect of colforsin on stroke volume index (mL/beat/m^2)

	Colforsin at Normal condition				Colforsin at Acidosis condition			
Variables	baseline	1st. Dose	2nd. Dose	3rd. Dose	baseline	1se. Dose	2nd. Dose	3rd. Dose
N	21	23	26	23	19	21	22	23
Sum total	914.39	1104.03	1284.89	1041.72	991.08	1127.47	1238.64	1347.28
Mean	43.54	48.00	49.42	45.29	52.16	53.69	56.30	58.58
Standard deviation	10.87	7.88	8.49	5.58	6.67	6.34	3.96	4.91
Standard error	2.37	1.64	1.67	1.16	1.53	1.38	0.84	1.02
Range	32.44	26.33	28.73	19.02	19.38	21.07	13.11	19.51
Minimum	26.96	38.27	36.52	36.67	40.62	44.25	49.14	51.32
Maximum	59.40	64.60	65.25	55.70	60.01	65.32	62.25	70.83
Median	40.82	47.62	51.87	45.03	54.18	55.83	56.54	56.96
25% quartile	37.26	41.24	39.94	40.62	44.95	46.87	52.99	55.22
75% quartile	54.62	51.52	54.18	49.12	57.00	58.75	59.78	61.07
Interval estimation of population mean	Reliability	95%			Reliability	95%		
Lower limit (t-distribution)	38.59	44.59	45.99	42.88	48.95	50.80	54.55	56.45
Upper limit (t-distribution)	48.49	51.41	52.85	47.70	55.37	56.58	58.06	60.70

Descriptive statistics value: The effect of dobutamine on stroke volume index (mL/beat/m^2)

	Dobutamine at Normal condition				Dobutamine at Acidosis condition			
Variables	baseline	1st. Dose	2nd. Dose	3rd. Dose	baseline	1se. Dose	2nd. Dose	3rd. Dose
N	18	18	18	18	19	19	18	20
Sum total	767.77	967.57	1028.82	963.30	971.86	1165.00	1145.90	1265.93
Mean	42.65	53.75	57.16	53.52	51.15	61.32	63.66	63.30
Standard deviation	4.88	6.85	6.99	3.79	5.48	9.27	6.58	6.51
Standard error	1.15	1.61	1.65	0.89	1.26	2.13	1.55	1.46
Range	17.86	24.01	22.81	13.87	16.73	32.14	23.26	21.55
Minimum	34.61	44.53	47.46	46.27	44.41	52.66	54.65	55.94
Maximum	52.47	68.54	70.27	60.13	61.15	84.80	77.91	77.49
Median	42.29	52.66	56.76	52.95	49.19	58.11	62.30	62.89
25% quartile	39.89	49.24	50.68	50.48	47.32	54.81	59.33	57.69
75% quartile	46.45	54.94	60.08	56.73	55.37	64.76	64.92	66.29
Interval estimation of population mean	Reliability	95%			Reliability	95%		
Lower limit (t-distribution)	40.23	50.35	53.68	51.63	48.51	56.85	60.39	60.25
Upper limit (t-distribution)	45.08	57.16	60.63	55.40	53.79	65.79	66.93	66.34

Descriptive statistics value: The effect of colforsin on systolic arterial pressure (mmHg)

	Colforsin at Normal condition				Colforsin at Acidosis condition			
Variables	baseline	1st. Dose	2nd. Dose	3rd. Dose	baseline	1se. Dose	2nd. Dose	3rd. Dose
N	21	23	26	23	19	21	22	23
Sum total	2019	2303	2401	1837	1770	1899	1844	1757
Mean	96.14	100.13	92.35	79.87	93.16	90.43	83.82	76.39
Standard deviation	16.06	13.82	10.59	8.63	5.64	6.77	15.48	17.97
Standard error	3.50	2.88	2.08	1.80	1.29	1.48	3.30	3.75
Range	46	33	26	23	17	15	41	52
Minimum	68	82	84	70	81	85	61	49
Maximum	114	115	110	93	98	100	102	101
Median	99	110	85	75	95	85	89	75
25% quartile	88	85	84	74	93	85	66	70
75% quartile	109	113	105	87.5	96	99	95	94
Interval estimation of population mean	Reliability	95%			Reliability	95%		
Lower limit (t-distribution)	88.83	94.15	88.07	76.14	90.44	87.35	76.96	68.62
Upper limit (t-distribution)	103.45	106.11	96.63	83.60	95.88	93.51	90.68	84.16

Descriptive statistics value: The effect of dobutamine on systolic arterial pressure (mmHg)

	Dobutamine at Normal condition				Dobutamine at Acidosis condition			
Variables	baseline	1st. Dose	2nd. Dose	3rd. Dose	baseline	1se. Dose	2nd. Dose	3rd. Dose
N	18	18	18	18	19	19	18	20
Sum total	1686	1758	1587	1434	1801	1912	1518	1461
Mean	93.67	97.67	88.17	79.67	94.79	100.63	84.33	73.05
Standard deviation	8.74	13.40	14.95	14.23	9.93	9.76	15.00	18.44
Standard error	2.06	3.16	3.52	3.35	2.28	2.24	3.54	4.12
Range	26	40	37	42	25	26	43	47
Minimum	82	76	65	60	80	86	62	54
Maximum	108	116	102	102	105	112	105	101
Median	93	98	94	81	91	106	82.5	66
25% quartile	87	89	72	66	86	89	75	58
75% quartile	99	109	102	88	105	108	99	93
Interval estimation of population mean	Reliability	95%			Reliability	95%		
Lower limit (t-distribution)	89.32	91.00	80.73	72.59	90.01	95.93	76.87	64.42
Upper limit (t-distribution)	98.02	104.33	95.60	86.74	99.57	105.34	91.79	81.68

Descriptive statistics value: The effect of colforsin on mean arterial pressure (mmHg)

	Colforsin at Normal condition				Colforsin at Acidosis condition			
Variables	baseline	1st. Dose	2nd. Dose	3rd. Dose	baseline	1se. Dose	2nd. Dose	3rd. Dose
N	21	23	27	23	19	20	21	22
Sum total	1438	1666	1769	1277	1261	1329	1271	1248
Mean	68.48	72.43	65.52	55.52	66.37	66.45	60.52	56.73
Standard deviation	11.73	11.07	8.14	9.93	8.37	7.02	11.88	13.01
Standard error	2.56	2.31	1.57	2.07	1.92	1.57	2.59	2.77
Range	36	33	22	34	29	21	36	41
Minimum	49	55	55	35	49	55	40	36
Maximum	85	88	77	69	78	76	76	77
Median	70	78	62	58	67	67	64	56.5
25% quartile	61	63	58	53.5	63.5	62	57	51
75% quartile	75	81	73.5	61.5	71	71.5	68	66
Interval estimation of population mean	Reliability	95%			Reliability	95%		
Lower limit (t-distribution)	63.14	67.65	62.30	51.23	62.34	63.16	55.12	50.96
Upper limit (t-distribution)	73.82	77.22	68.74	59.81	70.40	69.74	65.93	62.50

Descriptive statistics value: The effect of dobutamine on mean arterial pressure (mmHg)

	Dobutamine at Normal condition				Dobutamine at Acidosis condition			
Variables	baseline	1st. Dose	2nd. Dose	3rd. Dose	baseline	1se. Dose	2nd. Dose	3rd. Dose
N	21	23	27	23	19	20	21	22
Sum total	1438	1666	1769	1277	1261	1329	1271	1248
Mean	68.48	72.43	65.52	55.52	66.37	66.45	60.52	56.73
Standard deviation	11.73	11.07	8.14	9.93	8.37	7.02	11.88	13.01
Standard error	2.56	2.31	1.57	2.07	1.92	1.57	2.59	2.77
Range	36	33	22	34	29	21	36	41
Minimum	49	55	55	35	49	55	40	36
Maximum	85	88	77	69	78	76	76	77
Median	70	78	62	58	67	67	64	56.5
25% quartile	61	63	58	53.5	63.5	62	57	51
75% quartile	75	81	73.5	61.5	71	71.5	68	66
Interval estimation of population mean	Reliability	95%			Reliability	95%		
Lower limit (t-distribution)	63.14	67.65	62.30	51.23	62.34	63.16	55.12	50.96
Upper limit (t-distribution)	73.82	77.22	68.74	59.81	70.40	69.74	65.93	62.50

Descriptive statistics value: The effect of colforsin on diastolic arterial pressure (mmHg)

	Colforsin at Normal condition				Colforsin at Acidosis condition			
Variables	baseline	1st. Dose	2nd. Dose	3rd. Dose	baseline	1se. Dose	2nd. Dose	3rd. Dose
N	21	23	27	23	19	21	22	23
Sum total	1138	1344	1478	1155	960	986	972	944
Mean	54.19	58.43	54.74	50.22	50.53	46.95	44.18	41.04
Standard deviation	9.24	11.42	6.24	7.49	6.97	5.13	8.66	7.96
Standard error	2.02	2.38	1.20	1.56	1.60	1.12	1.85	1.66
Range	27	35	16	22	22	16	21	22
Minimum	40	40	44	42	37	39	31	28
Maximum	67	75	60	64	59	55	52	50
Median	56	62	58	49	52	47	47	41
25% quartile	46	47	48	44	48	45	34.25	39
75% quartile	61	65	59	52	55	51	51	47
Interval estimation of population mean	Reliability	95%			Reliability	95%		
Lower limit (t-distribution)	49.98	53.49	52.27	46.98	47.17	44.62	40.34	37.60
Upper limit (t-distribution)	58.40	63.38	57.21	53.46	53.89	49.29	48.02	44.48

Descriptive statistics value: The effect of dobutamine on diastolic arterial pressure (mmHg)

	Dobutamine at Normal condition				Dobutamine at Acidosis condition			
Variables	baseline	1st. Dose	2nd. Dose	3rd. Dose	baseline	1se. Dose	2nd. Dose	3rd. Dose
N	18	18	18	18	18	19	18	21
Sum total	984	951	864	792	931	1064	840	846
Mean	54.67	52.83	48.00	44.00	51.72	56.00	46.67	40.29
Standard deviation	9.35	8.11	6.26	4.60	6.37	8.16	5.99	6.34
Standard error	2.20	1.91	1.48	1.08	1.50	1.87	1.41	1.38
Range	27	24	18	15	22	24	17	18
Minimum	40	38	40	36	37	49	37	34
Maximum	67	62	58	51	59	73	54	52
Median	57	55.5	49.5	44	53	56	47.5	36
25% quartile	46	48	41	43	49	50	42	35
75% quartile	61	58	50	46	55	57	52	44
Interval estimation of population mean	Reliability	95%			Reliability	95%		
Lower limit (t-distribution)	50.02	48.80	44.89	41.71	48.55	52.06	43.69	37.40
Upper limit (t-distribution)	59.32	56.87	51.11	46.29	54.89	59.94	49.65	43.17

Descriptive statistics value: The effect of colforsin on right atrial pressure (mmHg)

	Colforsin at Normal condition				Colforsin at Acidosis condition			
Variables	baseline	1st. Dose	2nd. Dose	3rd. Dose	baseline	1se. Dose	2nd. Dose	3rd. Dose
N	21	23	27	23	20	21	22	23
Sum total	79	77	80	55	78	112	91	82
Mean	3.76	3.35	2.96	2.39	3.90	5.33	4.14	3.57
Standard deviation	1.37	1.30	1.22	1.03	0.91	0.48	0.64	0.73
Standard error	0.30	0.27	0.24	0.22	0.20	0.11	0.14	0.15
Range	4	3	3	3	3	1	2	2
Minimum	2	2	2	2	2	5	3	2
Maximum	6	5	5	5	5	6	5	4
Median	3	3	2	2	4	5	4	4
25% quartile	3	2	2	2	4	5	4	3
75% quartile	5	5	4	2	4	6	4.75	4
Interval estimation of population mean	Reliability	95%			Reliability	95%		
Lower limit (t-distribution)	3.14	2.79	2.48	1.94	3.47	5.11	3.85	3.25
Upper limit (t-distribution)	4.39	3.91	3.45	2.84	4.33	5.55	4.42	3.88

Descriptive statistics value: The effect of dobutamine on right atrial pressure (mmHg)

	Dobutamine at Normal condition				Dobutamine at Acidosis condition			
Variables	baseline	1st. Dose	2nd. Dose	3rd. Dose	baseline	1se. Dose	2nd. Dose	3rd. Dose
N	18	18	18	18	18	19	18	21
Sum total	54	54	51	45	90	88	81	96
Mean	3.00	3.00	2.83	2.50	5.00	4.63	4.50	4.57
Standard deviation	1.19	1.19	1.25	0.79	1.03	1.89	1.76	1.66
Standard error	0.28	0.28	0.29	0.19	0.24	0.43	0.41	0.36
Range	3	3	3	2	2	6	5	5
Minimum	2	2	2	2	4	1	2	2
Maximum	5	5	5	4	6	7	7	7
Median	2.5	2.5	2	2	5	5	4.5	4
25% quartile	2	2	2	2	4	4	3	4
75% quartile	4	4	4	3	6	6	6	6
Interval estimation of population mean	Reliability	95%			Reliability	95%		
Lower limit (t-distribution)	2.41	2.41	2.21	2.11	4.49	3.72	3.63	3.82
Upper limit (t-distribution)	3.59	3.59	3.45	2.89	5.51	5.54	5.37	5.33

Descriptive statistics value: The effect of colforsin on systemic vascular resistance index (dynes･sec･cm^{-5}/m^{2})

	Colforsin at Normal condition				Colforsin at Acidosis condition			
Variables	baseline	1st. Dose	2nd. Dose	3rd. Dose	baseline	1se. Dose	2nd. Dose	3rd. Dose
N	21	23	26	23	19	21	22	23
Sum total	124856	117306	87763	46310	63978	67646	53182	45202
Mean	5945.54	5100.26	3375.51	2013.50	3367.27	3221.23	2417.37	1965.33
Standard deviation	792.28	1479.79	787.66	467.81	945.97	962.85	522.16	520.44
Standard error	172.89	308.56	154.47	97.55	217.02	210.11	111.33	108.52
Range	2546.51	4635.85	2574.65	1405.07	2991.29	2903.48	1640.50	1523.93
Minimum	4605	2772	1951	1324	2451	2291	1842	1393
Maximum	7152	7407	4526	2729	5442	5195	3483	2917
Median	6136.15	5270.09	3542.52	2022.24	3086.42	2942.24	2220.30	1761.01
25% quartile	5153.47	3564.34	2718.95	1548.26	2685.91	2652.03	2048.09	1599.84
75% quartile	6540.32	6043.72	3917.05	2452.81	3580.47	3115.75	2796.21	2525.47
Interval estimation of population mean	Reliability	95%			Reliability	95%		
Lower limit (t-distribution)	5584.89	4460.35	3057.36	1811.20	2911.33	2782.95	2185.85	1740.27
Upper limit (t-distribution)	6306.18	5740.17	3693.65	2215.79	3823.22	3659.51	2648.88	2190.38

Descriptive statistics value: The effect of dobutamine on systemic vascular resistance index (dynes･sec･cm^{-5}/m^2)

	Dobutamine at Normal condition				Dobutamine at Acidosis condition			
Variables	baseline	1st. Dose	2nd. Dose	3rd. Dose	baseline	1se. Dose	2nd. Dose	3rd. Dose
N	18	18	18	18	18	19	18	20
Sum total	104727	90234	46206	35264	64296	52800	34141	30556
Mean	5818.15	5013.01	2567.02	1959.11	3572.01	2778.95	1896.70	1527.80
Standard deviation	2037.51	2985.44	926.84	508.07	1128.88	883.44	490.14	423.98
Standard error	480.25	703.67	218.46	119.75	266.08	202.67	115.53	94.80
Range	6572.92	8783.02	2715.64	1437.13	3029.10	2668.39	1579.27	1302.67
Minimum	3611	2035	1546	1420	2501	1651	1303	924
Maximum	10184	10818	4261	2858	5530	4319	2882	2227
Median	5418.60	3881.67	2395.79	1848.48	3038.22	2391.03	1859.08	1571.28
25% quartile	4411.51	2559.44	1730.29	1515.49	2684.79	2136.00	1522.63	1143.26
75% quartile	6178.81	7056.68	3146.86	2232.80	4919.00	3429.93	2034.29	1809.81
Interval estimation of population mean	Reliability	95%			Reliability	95%		
Lower limit (t-distribution)	4804.92	3528.38	2106.11	1706.45	3010.64	2353.15	1652.95	1329.37
Upper limit (t-distribution)	6831.38	6497.63	3027.92	2211.76	4133.39	3204.75	2140.44	1726.23

Descriptive statistics value: The effect of colforsin on pulmonary artery occlusion pressure (mmHg)

	Colforsin at Normal condition				Colforsin at Acidosis condition			
Variables	baseline	1st. Dose	2nd. Dose	3rd. Dose	baseline	1se. Dose	2nd. Dose	3rd. Dose
N	21	23	27	23	18	21	22	23
Sum total	94	92	91	77	197	206	191	193
Mean	4.48	4.00	3.37	3.35	10.94	9.81	8.68	8.39
Standard deviation	0.75	0.60	0.69	1.50	1.00	2.32	1.39	0.94
Standard error	0.16	0.13	0.13	0.31	0.24	0.51	0.30	0.20
Range	2	2	2	4	3	8	4	2
Minimum	4	3	3	2	9	6	7	7
Maximum	6	5	5	6	12	14	11	9
Median	4	4	3	3	11	9	9	9
25% quartile	4	4	3	2	10.25	9	7.25	7
75% quartile	5	4	3.5	3	12	11	9.75	9
Interval estimation of population mean	Reliability	95%			Reliability	95%		
Lower limit (t-distribution)	4.13	3.74	3.10	2.70	10.45	8.76	8.06	7.98
Upper limit (t-distribution)	4.82	4.26	3.64	3.99	11.44	10.86	9.30	8.80

Descriptive statistics value: The effect of dobutamine on pulmonary artery occlusion pressure (mmHg)

	Dobutamine at Normal condition				Dobutamine at Acidosis condition			
Variables	baseline	1st. Dose	2nd. Dose	3rd. Dose	baseline	1se. Dose	2nd. Dose	3rd. Dose
N	18	18	18	18	18	19	18	21
Sum total	84	87	84	78	177	200	180	208
Mean	4.67	4.83	4.67	4.33	9.83	10.53	10.00	9.90
Standard deviation	1.28	1.62	0.77	1.28	2.33	1.81	1.78	1.45
Standard error	0.30	0.38	0.18	0.30	0.55	0.41	0.42	0.32
Range	3	4	2	3	7	6	4	4
Minimum	3	3	4	3	6	7	8	8
Maximum	6	7	6	6	13	13	12	12
Median	5	5	4.5	4	10.5	11	10	9
25% quartile	3	3	4	3	8	10	8	9
75% quartile	6	6	5	6	11	11	12	11
Interval estimation of population mean	Reliability	95%			Reliability	95%		
Lower limit (t-distribution)	4.03	4.03	4.29	3.70	8.67	9.66	9.11	9.25
Upper limit (t-distribution)	5.30	5.64	5.05	4.97	10.99	11.40	10.89	10.56

Descriptive statistics value: The effect of colforsin on mean pulmonary artery pressure (mmHg)

	Colforsin at Normal condition				Colforsin at Acidosis condition			
Variables	baseline	1st. Dose	2nd. Dose	3rd. Dose	baseline	1se. Dose	2nd. Dose	3rd. Dose
N	21	23	26	23	19	21	22	23
Sum total	224	248	312	307	321	395	429	462
Mean	10.67	10.78	12.00	13.35	16.89	18.81	19.50	20.09
Standard deviation	0.73	1.73	2.83	2.93	3.77	0.40	0.96	1.12
Standard error	0.16	0.36	0.55	0.61	0.86	0.09	0.21	0.23
Range	2	5	8	8	12	1	3	3
Minimum	10	8	8	10	9	18	18	19
Maximum	12	13	16	18	21	19	21	22
Median	11	10	12	12	18	19	19	20
25% quartile	10	10	10	11	17	19	19	19
75% quartile	11	12	14	17	19	19	20	21
Interval estimation of population mean	信頼度	95%			信頼度	95%		
Lower limit (t-distribution)	10.33	10.03	10.86	12.08	15.08	18.63	19.07	19.60
Upper limit (t-distribution)	11.00	11.53	13.14	14.62	18.71	18.99	19.93	20.57

Descriptive statistics value: The effect of dobutamine on mean pulmonary artery pressure (mmHg)

	Dobutamine at Normal condition				Dobutamine at Acidosis condition			
Variables	baseline	1st. Dose	2nd. Dose	3rd. Dose	baseline	1se. Dose	2nd. Dose	3rd. Dose
N	18	18	18	18	19	19	18	20
Sum total	201	261	330	354	355	451	450	534
Mean	11.17	14.50	18.33	19.67	18.68	23.74	25.00	26.70
Standard deviation	1.92	2.12	1.53	2.35	1.38	1.41	1.68	2.25
Standard error	0.45	0.50	0.36	0.55	0.32	0.32	0.40	0.50
Range	4	6	4	7	4	4	5	6
Minimum	9	11	17	16	16	21	22	23
Maximum	13	17	21	23	20	25	27	29
Median	11.5	15	18	20	19	24	25.5	27
25% quartile	9	13	17	18	18	23	24	25
75% quartile	13	16	19	21	20	25	26	29
Interval estimation of population mean	Reliability	95%			Reliability	95%		
Lower limit (t-distribution)	10.21	13.45	17.57	18.50	18.02	23.06	24.16	25.65
Upper limit (t-distribution)	12.12	15.55	19.10	20.84	19.35	24.42	25.84	27.75

Descriptive statistics value: The effect of colforsin on pulmonary vascular resistance index (dynes･sec･cm^{-5}/m^2)

	Colforsin at Normal condition				Colforsin at Acidosis condition			
Variables	baseline	1st. Dose	2nd. Dose	3rd. Dose	baseline	1se. Dose	2nd. Dose	3rd. Dose
n	21	23	26	23	19	21	22	23
Sum total	12751	11312	11822	8725	7506	9819	10636	10176
Mean	607.19	491.81	454.68	379.37	395.03	467.56	483.47	442.44
Standard deviation	239.48	155.13	163.31	136.95	79.09	156.57	134.24	74.80
Standard error	52.26	32.35	32.03	28.56	18.14	34.17	28.62	15.60
Range	791.65	502.10	558.95	419.57	255.07	500.57	436.17	253.77
Minimum	346.10	341.12	228.31	172.84	272.32	241.55	271.32	298.31
Maximum	1137.75	843.21	787.26	592.40	527.39	742.12	707.48	552.08
Median	520.49	412.12	432.33	393.02	408.16	457.25	515.88	457.33
25% quartile	456.97	397.95	327.67	280.46	322.73	351.03	354.72	401.61
75% quartile	657.44	544.64	516.06	502.70	451.77	515.46	542.05	493.46
Interval estimation of population mean	Reliability	95%			Reliability	95%		
Lower limit (t-distribution)	498.18	424.73	388.72	320.15	356.91	396.29	423.95	410.09
Upper limit (t-distribution)	716.20	558.89	520.65	438.59	433.15	538.83	542.99	474.79

Descriptive statistics value: The effect of dobutamine on pulmonary vascular resistance index (dynes・sec・cm^{-5}/m^2)

	Dobutamine at Normal condition				Dobutamine at Acidosis condition			
Variables	baseline	1st. Dose	2nd. Dose	3rd. Dose	baseline	1se. Dose	2nd. Dose	3rd. Dose
n	18	18	18	18	19	19	18	20
Sum total	10259	11819	9887	9544	9243	10220	9101	10396
Mean	569.94	656.62	549.30	530.22	486.48	537.89	505.63	519.81
Standard deviation	136.76	243.52	133.90	133.69	131.19	173.36	148.54	136.34
Standard error	32.24	57.40	31.56	31.51	30.10	39.77	35.01	30.49
Range	399.18	693.88	396.74	365.63	444.64	465.12	422.90	434.22
Minimum	346.28	337.71	409.48	338.41	294.27	310.08	328.98	308.17
Maximum	745.46	1031.59	806.22	704.04	738.92	775.19	751.88	742.39
Median	575.55	580.49	494.40	524.93	478.97	516.68	487.47	533.02
25% quartile	471.34	499.04	445.44	416.67	391.03	371.26	365.90	419.52
75% quartile	707.84	937.10	619.84	665.92	525.40	726.74	629.39	615.71
Interval estimation of population mean	Reliability	95%			Reliability	95%		
Lower limit (t-distribution)	501.93	535.52	482.71	463.73	423.25	454.34	431.77	456.00
Upper limit (t-distribution)	637.96	777.71	615.89	596.70	549.71	621.45	579.50	583.62
